# Development and validation of a necroptosis-related gene prognostic score to predict prognosis and efficiency of immunotherapy in gastric cancer

**DOI:** 10.3389/fimmu.2022.977338

**Published:** 2022-08-26

**Authors:** Yan Xia, Rongzheng Zhang, Mingzhu Wang, Jiaqi Li, Jianming Dong, Kaitong He, Ting Guo, Xiaomei Ju, Jiaqiu Ru, Shuyun Zhang, Yihua Sun

**Affiliations:** ^1^ Department of Clinical Laboratory, Harbin Medical University Cancer Hospital, Harbin, China; ^2^ Scientific Research Center, the Second Affiliated Hospital of Harbin Medical University, Harbin, China

**Keywords:** necroptosis gene, gastric cancer, bioinformatics analysis, prognosis, tumor microenvironment

## Abstract

Necroptosis is a novel type of regulated cell death that is intimately associated with a variety of tumors. However, how necroptosis affects the identification of gastric cancer (GC) remains unclear. Here we seek to find new potential necroptosis-related biomarkers to predict GC prognosis and immunotherapy effect. We used Cox analysis to obtain shared prognostic markers related to necroptosis from five datasets (TCGA and four GEO datasets). Then, a necroptosis-related gene prognostic score (NRGPS) system was constructed using LASSO Cox regression, NRGPS consisting of three necroptosis-related mRNAs (*AXL*, *RAI14*, and *NOX4*) was identified, 31 pairs of GC and adjacent normal tissues from the Second Hospital of Harbin Medical University were collected and Real-Time Quantitative PCR (RT-qPCR) was used to detect the relative expression levels of the three necroptosis-related mRNAs, and external validation was performed on four GEO datasets (GSE84437, GSE26901, GSE62254 and GSE15459). In this study, Overall survival (OS) in the high-NRGPS group was significantly lower than in the low-NRGPS group. Cox regression analyses showed that NRGPS was an independent prognostic variable. Tumor-mutation-burden (TMB), tumor microenvironment (TME), microsatellite instability (MSI), and Tumor Immune Dysfunction and Exclusion (TIDE) scoring were used as predictors of the immunotherapy response. A cancer-friendly immune microenvironment, a high TIDE score, a low TMB, and a low MSI were all characteristics of the high-NRGPS group, and they all consistently showed that the issues seen there are related to immune escape in GC. The combination of three candidate genes may be an effective method for diagnostic assessment of GC prognosis and immunotherapy efficacy.

## Introduction

Over one million new cases of gastric cancer (GC) and close to 80,000 GC-related fatalities were reported in 2020, making it one of the most prevalent cancers in the world (1). Immunotherapy has improved the treatment strategy for several types of malignancies, including GC, as ICB has the potential to induce durable immune responses in different types of cancers (2, 3). However, only one-third of individuals with the majority of malignancies benefit with checkpoint inhibitors (4). This resistance is usually through a well-established mechanism of immune evasion of the cancer cells, leading to the spread of the disease (5). GC prognosis is currently based on the Lauren and World Health Organization classification as well as the Tumor-Node-Metastasis (TNM) staging system (6). However, patients in the same stage of cancer may demonstrate significantly different prognoses. Although many patients have similar clinical characteristics and associated treatment options, outcomes may differ markedly. Thus, to improve the prognosis of GC patients, it is necessary to investigate trustworthy biomarkers that can reliably predict prognosis and identify targets for prospective treatment.

Necroptosis is a new type of programmed necrotic cell death, which is regulated differently from apoptosis. The necroptosis signaling pathway includes activated receptor-interacting protein kinases (RIPKs) as well as mixed lineage kinase domain-like pseudokinases (MLKL) (7). Necroptosis induces strong cross-priming of anti-tumor CD8^+^ T cells by releasing damage-associated molecular patterns, thereby suppressing tumor progression (8). A recent study showed that nano-vaccines that mimic necrotic cancer cells could enhance immunity in mice through the proliferation of NKG2D^+^ natural killer cells and CD8^+^ T cells, ultimately enhancing the anti-tumor effects (9). Another study showed that the activation of RIPK3 results in a reliable derepression of tripartite motif-containing 28 in cancer cells, thereby inducing increased production of immunostimulatory cytokines in the tumor microenvironment (TME), thus promoting potent cytotoxic antitumor immunity (10).

Recent basic experiments and bioinformatics analyses have revealed that MLKL mRNA expression levels are dramatically lower in GC than normal tissue and that GC patients with low MLKL expression have a poorer prognosis compared to normal tissues (11). Another study found that astaxanthin caused AGS death in GC cell lines by inducing increased NADPH oxidase activity, ROS production, and phosphorylation of RIP1/RIP3/MLKL (12). This indicates that necroptosis is related to the prognosis and treatment of GC. Necroptosis is a new target for cancer therapy, considering its critical role in cancer biology (13). The quality and quantity of innate necroptosis-centered immune cell rely on the inflammatory background, tissue type, and other individual circumstances (7). However, only a small number of research have examined the connection between necroptosis-related genes (NRGs) and the prognosis and therapy of GC (14, 15).

In this study, The Cancer Genome Atlas (TCGA) was searched to develop prognostic NRGs markers for GC. We identified three mRNA markers with reliable prognostic expression and experimentally validated NRGPS using RT-qPCR assay for GC and adjacent normal tissues to detect their relative mRNA level, as well as independent external validation using the GSE84437, GSE26901, GSE62254 and GSE15459 datasets. It was finally demonstrated that NRGPS could be used as an independent prognostic indicator for GC. Our aim is to fully investigate the expression profile of necroptosis and the predicted effect of immunotherapy treatment, thus identifying potential targets for the treatment of GC. We hope to our study enable the improved stratification of GC patients, thus facilitating personalized treatment decisions.

## Materials and methods

### Clinical specimen collection

Thirty-one pairs of GC and healthy tissue samples from cancer were gathered and frozen in liquid nitrogen at the Second Hospital of the Harbin Medical University.All specimens were judged by experienced pathologists and informed consent and permission was obtained from the Medical Ethics Committee of the Second Affiliated Hospital of Harbin Medical University. Detailed clinicopathological data of the patients are shown in **Supplementary Table S1**.

### Acquisition of Information of patients with GC 

**Figure 1:**
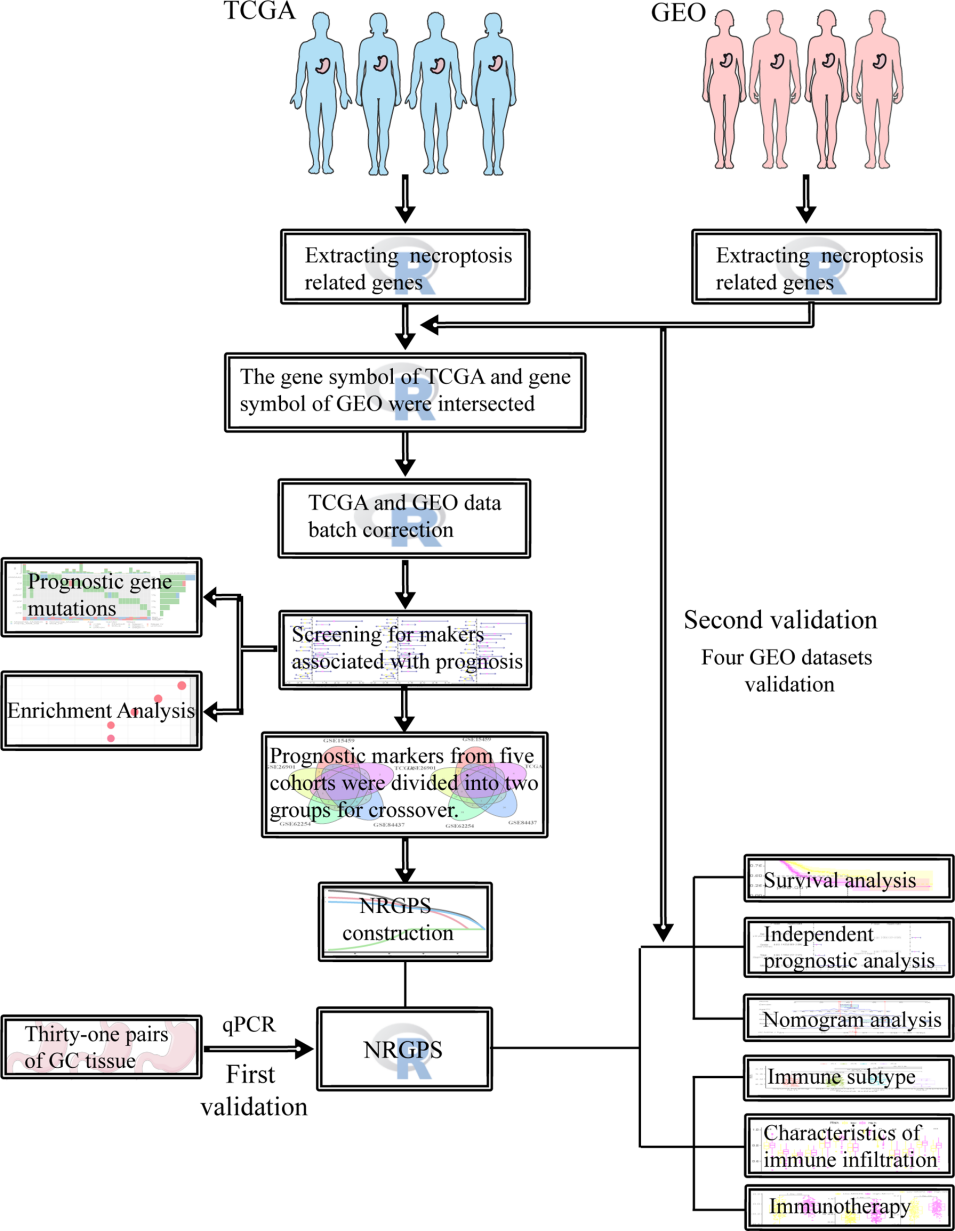
Flowchart of this study.

The flow chart of this study is shown in **Figure 1**. The RNA-Seq data, clinical information, and somatic mutations of GC patients (375 tumor samples, 32 normal samples) were obtained from TCGA (https://portal.gdc.cancer.gov/). The external validation cohorts GSE84437 (n = 433), GSE26901 (n = 109), GSE62254 (n = 300) and GSE15459 (n = 192) were obtained from GEO (https://www.ncbi.nlm.nih.gov/geo/). In addition, GSE29272 (134 pairs) and GSE63089 (45 pairs) with adjacent normal tissues as controls, and the melanoma cohort (GSE78220) with anti-PD-1 checkpoint inhibition therapy were similarly derived from GEO. Gene Expression Profiling Interactive Analysis (GEPIA; http://gepia.cancer-pku.cn/) contains data from TCGA and Genotype-Tissue Expression (GTEx). 583 NRGs sets were derived from GeneCards (https://www.genecards.org/). After 12 non-coding RNAs were removed, 571 necroptosis-related mRNAs were obtained.

### Identication of prognosis markers

First, we extracted necroptosis-related gene expression data from TCGA and GEO cohorts. Then, the non-technical batch effect between TCGA and GEO data was corrected before analysis by using the “sva” R package. In addition, we also intersect the gene symbols of TCGA cohort with GEO cohorts to ensure that the genes obtained from subsequent analysis are shared by the five data sets. Next, univariable Cox regression analysis was performed for each cohort to further screen for potential prognostic markers. The venn diagrams were drawn using the “VennDiagram” R package to show common prognostic markers in all five cohorts.

### Construction and validation of the necroptosis-related gene prognostic score system

The Least Absolute Shrinkage and Selection Operator (LASSO) Cox regression method was used to create the NRGPS system, and tenfold cross validation was used to estimate the ideal coefficients based on the partial likelihood deviance. To determine the patient risk scores, regression coefficients of the genes and matching mRNA expression levels were employed.The formula for calculating NRGPS is as follows:


$$NRGPS=\sum_{i=1}^{\text{n}}\text{coef}(\text{gene}i)*\text{expr}(\text{gene}i)$$

The median NRGPS was used to separate the high-NRGPS group from the low-NRGPS group. Using the Kaplan-Meier curve, the survival status of both the high-NRGPS and low-NRGPS groups was examined. The prognostic significance of the created marker for predicting survival was evaluated using a time-dependent receiver operating characteristic (ROC) curve analysis. The independent prognostic significance of NRGPS was assessed using univariable and multivariable Cox regression analysis.At the same time, we performed the same analysis on four GEO cohorts to verify. The above analysis was performed using the “survminer”, “survival”, and “timeROC” R packages.

### Detection of NRGs mRNA expression levels in GC tissues by RT-qPCR

Total RNA was extracted from GC and Normal using Trizol reagent (SM129-02, Sevenbio, China); cDNA was synthesized using a reverse transcription kit (1119ES60, Yeasen, China); SYBR Green Master Mix kit (11184ES03, Yeasen, China) and RT-qPCR instrument (SLAN-96p Shanghai hongshi, China) were used for RT-qPCR, glyceraldehyde-3phosphate dehydrogenase (GAPDH) was used as an internal control, and three NRGs quantification was based on the 2^-ΔΔCt^ method, and the primer sequences are shown in **Supplementary Table 2**.

### Function enrichment analysis

The “clusterProfiler”, “enrichplot”, and “ggplot2” R packages were used to conduct the Gene Ontology (GO) and Kyoto Encyclopedia of Genes and Genomes (KEGG) pathway studies (16, 17). Then, we performed a Gene Set Enrichment Analysis (GSEA) to investigate potential biological courses.c2.cp.kegg.v7.4.symbols.gmt was chosen as the reference file, and meaningful biological processes and pathways were enriched to FDR< 0.05. The protein-protein interactions (PPI) of prognostic markers were shown using a string database (https://www.string-db.org/).

### Development of nomogram based on NRGPS and clinical features

Nomogram was created to predict the survival probability of gastric cancer patients at 3 and 5 years. The ROC curve of nomogram is drawn to evaluate its accuracy. The “rms”,”regplot”, “survival”, “survminer” and “timeROC” R packages were used for the above analysis.

### Correlation analysis to identify an association between NRGPS and immune typing

Based on immune typing files (Subtype_Immune_Model_Based.txt), GC patients were classified into six subtypes (18). Then observe whether there are differences in NRGPS between patients with different immune subtypes.

### Immune cell infiltration analysis

First, on the TCGA cohort, it was done using single sample gene set enrichment analysis (ssGSEA).The relative abundance of 16 immune cell infiltrates and the activity of 13 immunological-related pathways in the TME were represented by enrichment scores.Next, the correlation between three candidate genes and NRGPS and immune cells and pathways was analyzed. The relationship between immune cell infiltration and GC patients’ survival time was further analyzed. Immune cell infiltration and NRGPS were combined to perform a survival analysis of the GC patients. The absolute mode of cell type identification by estimating relative subsets of RNA transcripts (CIBERSORT-ABS) technique was utilized to confirm the immune cells’ invasion of TME. Finally, to confirm the relationship between the three candidate genes and macrophages, estimating the proportion of Immune and cancer cells (EPIC), tumor immune estimation resource (TIMER), comprehensive bioinformatic deconvolution (xCELL), and microenvironment cell populations- counter (MCPCOUNTER) techniques were employed. The data of CIBERSORT-ABS in TCGA cohort comes from TIMER (http://timer.cistrome.org/). The EPIC, TIMER, xCELL and MCPCOUNTER algorithms are implemented based on the TIMER website.

### Prediction of immunotherapy effect

Based on the somatic mutation data downloaded from TCGA, the number of mutations in each gene in the sample was first counted to analyze the mutation status in the high- and low-NRGPS groups. Then, the “limma”, “ggplot2”, “ggpubr”, and “ggExtra” R packages were used for the following: analyze the difference in the TMB of the high- and low-NRGPS groups; identify a correlation between NRGPS and TMB; Then, The link between TMB and prognosis was assessed using the TMB value and the accompanying survival data. Finally, NRGPS and TMB were combined to perform a survival analysis of the GC samples.

We plotted the percentage histogram of microsatellite instability (MSI) states of GC patients in the high- and low-NRGPS groups. Then, the boxplot is drawn to show the NRGPS differences of different MSI groups. The MSI status of GC patients in the TCGA cohort was downloaded from The Cancer Immunome Atlas (TCIA; https://tcia.at/home) database.

We further analyzed the correlation between the NRGPS and immune checkpoints and plotted the correlation plot using the “corrplot” R package. Then, the expression of immune checkpoints between high- and low-NRGPS groups was compared.

Immune escape and treatment were evaluated by subjecting the high- and low-NRGPS groups to tumor immune dysfunction and exclusion (TIDE; http://tide.dfci.harvard.edu/). The TIDE score, dysfunction score, and immune exclusion score were obtained from TIDE.

The GSE78220 cohort was used to verify the effect of NRGPS in predicting immunotherapy. We divided the GSE78220 cohort into high- and low-NRGPS groups according to the median NRGPS, and plotted the survival curve and ROC curve. Then, the boxplot was drawn to compare the NRGPS of patients who responded and did not respond to the treatment. In addition, we drew a histogram and compared whether there was a difference between the proportion of patients who responded and did not respond to the treatment in the high- and low-NRGPS groups.

### Statistical analysis

Statistical analysis of this study was conducted by R (Version 4.1.2) and SPSS software (version 25.0). Including Cox regression analysis, Lasso analysis, Kaplan-Meier survival analysis, ROC curve analysis, independent prognostic analysis, functional analysis, nomogram analysis, immune cell infiltration analysis, correlation analysis, TMB analysis and TIDE analysis. To compare the differences between the two groups of data, we used the Wilcoxon test. The Spearman method was used for correlation analysis. RT-qPCR data did not conform to normal distribution, and non-parametric Wilcoxon’s matched-pairs test was conducted. The paired samples of GSE29272 and GSE63089 also used non-parametric Wilcoxon’s matched-pairs test. Fisher’s exact test was used to determine the proportion of patients who responded to treatment in the high- and low-NRGPS groups of the GSE29272 cohort. The R packages and statistical methods used by GEO validation cohorts are consistent with TCGA. *p<* 0.05 was considered statistically significant.

## Results

### Identification of prognosis markers and functional enrichment analysis

**Figure 2:**
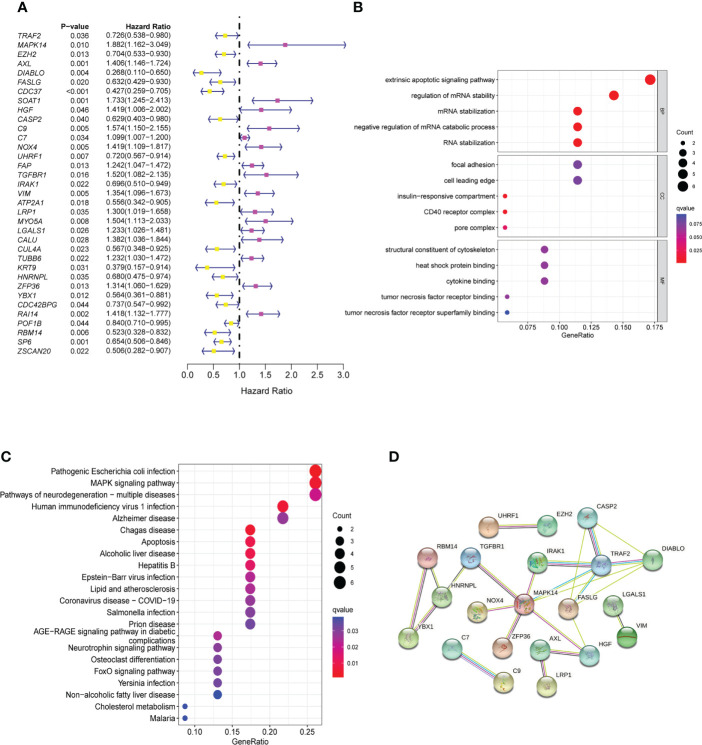
Identification and functional enrichment analysis of prognostic markers. **(A)** The forestmap of 35 prognostic markers of GC patients in the TCGA cohort. **(B)** The GO enrichment analysis of the prognostic markers. **(C)** The KEGG pathway analysis of the prognostic markers. **(D)** The PPI network was constructed through 35 prognostic markers. The interaction score was set to 0.4. GC, Gastric cancer; GO, Gene Ontology; KEGG, Kyoto Encyclopedia of Genes and Genomes; PPI, protein-protein interaction.

To explore the prognostic value of the NRGs, we conducted a univariable Cox regression analysis for GC patients in TCGA cohort and GEO cohorts. The results showed that 35 NRGs were significantly associated with GC prognosis in TCGA cohort (**Figure 2A**). NRGs of the four GEO cohorts significantly associated with prognosis are presented in **Supplementary Table 3**. To learn more about the function of prognostic markers in GC, we conducted GO and KEGG pathway enrichment analyses. The GO analysis revealed that prognostic indicators were enriched in necroptosis development and maintenance,such as “extrinsic apoptotic signaling pathway”, “regulation of mRNA stability”, and “mRNA stabilization” (**Figure 2B**). The most abundant pathways in the KEGG analysis were related to “MAPK signaling pathway”, “apoptosis” and “pathogenic escherichia coli infection” (**Figure 2C**). Together, these findings are associated with programmed cell death and tumor progression. The PPI network of 21 nodes and 24 edges showed a complex relationship between these prognostic markers in GC (**Figure 2D**).

### Development and validation of the NRGPS system

**Figure 3:**
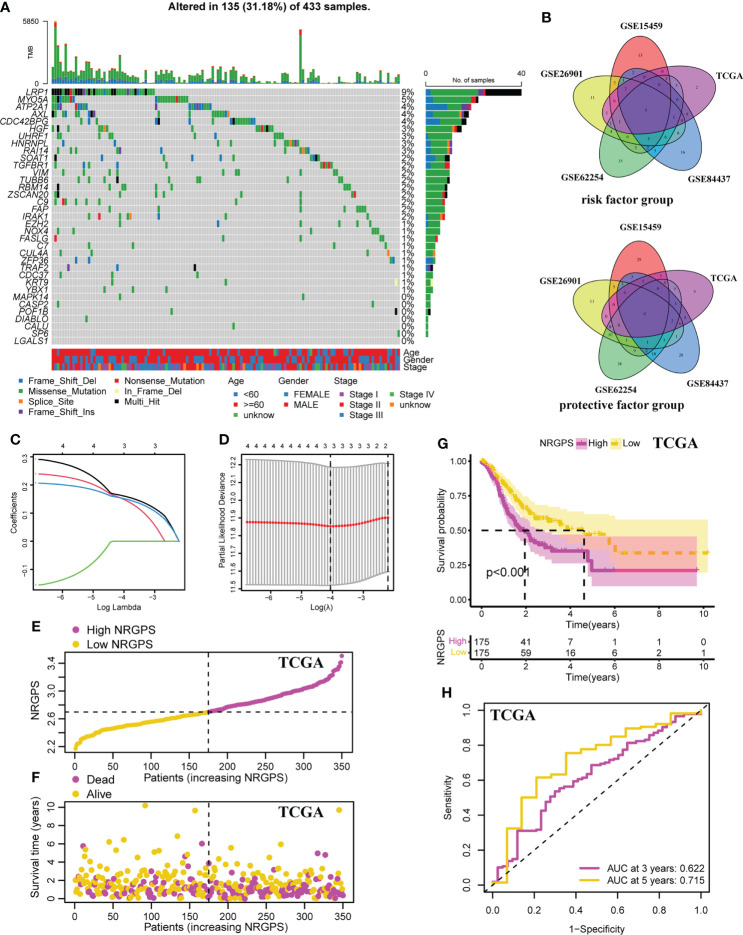
Development of the NRGPS System. **(A)** The waterfall plot of the prognostic associated genes mutations. **(B)** The venn plots of intersection of prognostic markers in five cohorts were shown. **(C)** LASSO coefficient profiles of prognostic markers. **(D)** The tuning parameters were cross-validated in the LASSO model. **(E)** Distribution of NRGPS in TCGA cohort. **(F)** Survival status in the high-NRGPS and low-NRGPS groups of TCGA cohort. **(G)** Kaplan-meier survival analysis in TCGA cohort. **(H)** The ROC curve of TCGA cohort verifies the prediction ability of this prediction model. LASSO, Least Absolute Shrinkage and Selection Operator. NRGPS, necroptosis-related gene prognostic score.

To better understand the mechanism by which these 35 NRGs affected the prognosis of the GC patients, we further analyzed their somatic mutation status in GC samples. The result indicated that 135 of 433 (31.18%) GC samples demonstrated genetic mutations. The missense mutation was the most common type of variation (**Figure 3A**).

In order to ensure the accuracy of prognostic markers, we divided prognostic markers into risk factor (HR > 1) and protective factor (HR< 1) groups according to HR values obtained by univariable Cox regression analysis, and then divided prognostic markers from five cohorts into two groups for intersection. Finally, in the risk factor group, there were five intersection genes (*TUBB6*, *LGALS1*, *AXL*, *RAI14*, and *NOX4*) in five cohorts (**Figure 3B**).

**Figure 4:**
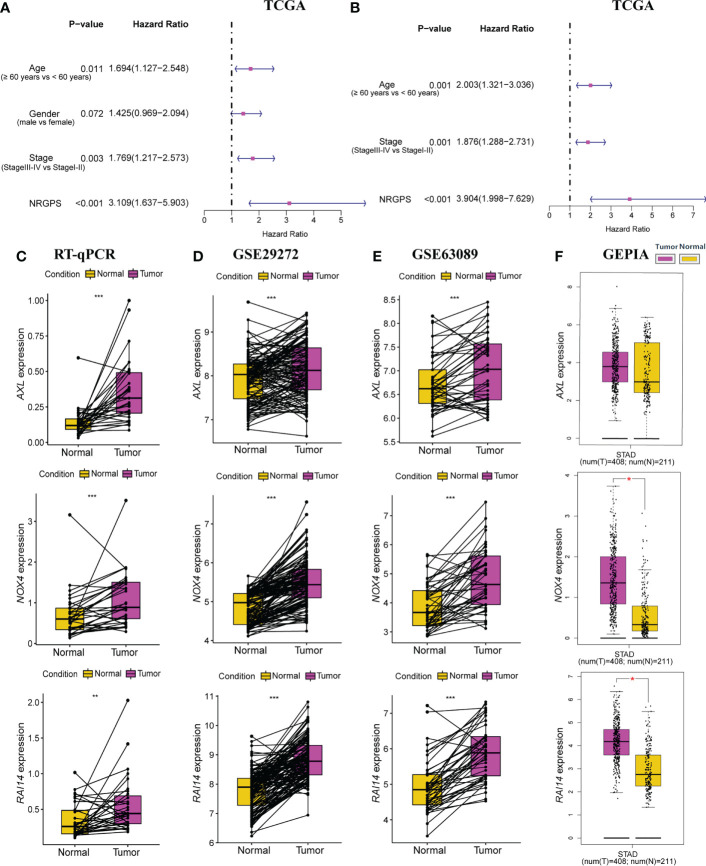
Independent prognostic analysis of prognostic model in TCGA cohort was performed. **(A)** The forestplot of univariable Cox regression analysis of NRGPS and clinical characteristics in TCGA cohort. **(B)** The forestplot of multivariable Cox regression analysis of NRGPS and clinical characteristics in TCGA cohort. **(C)** RT-qPCR was used to detect the relative mRNA levels of three NRGs in GC and adjacent normal tissues. **(D)** Relative mRNA levels of three NRGs in GC and adjacent normal tissues in GSE29272 dataset. **(E)** Relative mRNA levels of three NRGs in GC and adjacent normal tissues in GSE63089 dataset. **(F)** Relative mRNA levels of three NRGs in GC and adjacent normal tissues in GEPIA dataset. NRGPS, necroptosis-related gene prognostic score; NRG, necroptosis-related genes; GEPIA, Gene Expression Profiling Interactive Analysis. *p < 0.05, **p < 0.01, ***p < 0.001.

Lasso Cox regression was performed on the five risk factor genes to obtain the NRGs markers (**Figures 3C, D**). Then, an NRGPS was constructed as follows: score = 0.162 × expression quantity of *AXL* + 0.152 × expression quantity of *NOX4* + 0.153 × expression quantity of *RAI14*), with the median NRGPS of TCGA cohort as the critical value. In addition, scatter plots indicated that GC patients in the high-NRGPS group had a higher proportion of death and shorter survival time than those in the low-NRGPS group (**Figures 3E, F**). The Kaplan–Meier curve indicated that the overall survival (OS) of the high-NRGPS group was shorter than that of the low-NRGPS group (**Figure 3G**). The area under the curve (AUC) in the time-dependent ROC analysis was 0.622 at 3 years and 0.715 at 5 years (**Figure 3H**), indicating great specificity and sensitivity of the NRGPS in predicting the OS. To assess the value of NRGPS as a standalone prognostic marker, we performed univariable and multivariable Cox analyses to identify its correlation with age (≥ 60 years vs< 60 years), gender (male vs female), Stage (StageIII-IV vs StageI-II) in the GC patients in TCGA cohorts. Univariable Cox regression analysis showed that the NRGPS was associated with GC prognosis in TCGA cohort (**Figure 4A**). Multivariable Cox regression confirmed that the NRGPS was an independent predictor of survival after correcting for other clinical confounding factors in TCGA cohort (**Figure 4B**). In addition, we also performed a stratified analysis to assess whether NRGPS is applicable to patients with different clinical characteristics (**Figure S1A**).

### Validation of 3 NRGs in GC tissue specimens

RT-qPCR was used to detect the relative mRNA level of 3 NRGs in 31 pairs of GC and adjacent normal tissues, Results from RT-qPCR revealed that the expression levels of *AXL*, *RAI14*, and *NOX4* were higher in GC tissues than in nearby normal tissues next to cancer (**Figure 4C**), further demonstrating from the perspective of basic experiments that the reliability of NRGPS for determining GC prognosis. In addition, we also added three data sets and obtained the same trend results (**Figures 4D–F**).

### GEO external verification of NRGPS

**Figure 5:**
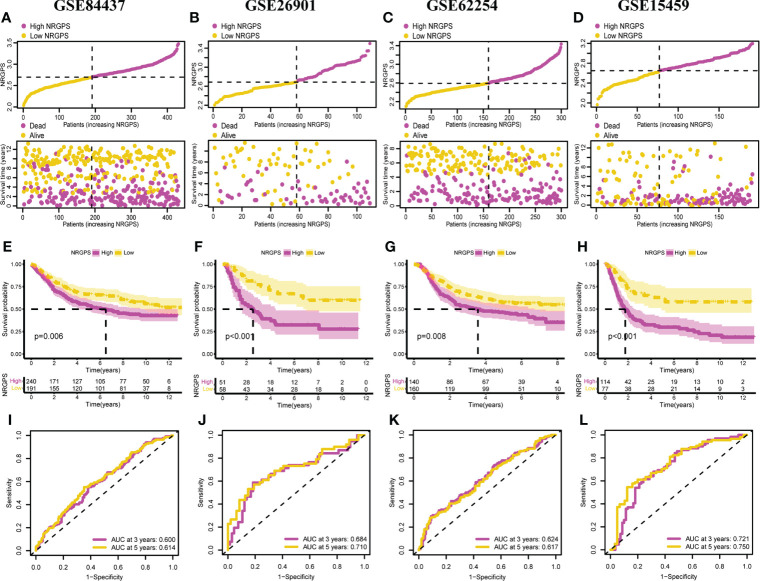
Validation of the NRGPS System. Distribution of NRGPS and survival status in the high-NRGPS and low-NRGPS groups in **(A)** GSE84437, **(B)**  GSE62901, **(C)** GSE62254, **(D)** GSE15459. Kaplan-Meier survival analysis in **(E)** GSE84437, **(F)** GSE62901, **(G)** GSE62254, **(H)** GSE15459. The ROC analysis in **(I)** GSE84437, **(J)** GSE62901, **(K)** GSE62254, and **(L)** GSE15459 verifies the prediction ability of this prediction model. NRGPS, necroptosis-related gene prognostic score; GC, Gastric cancer; ROC, receiver operating characteristic.

To evaluate the stability of NRGPS—constructed from TCGA cohort—as a prognostic biomarker for GC, four independent GEO data sets were used for validation. Then, according to the median NRGPS of TCGA cohort, 240 GC patients were included in the high-NRGPS group and 191 GC patients were included in the low-NRGPS group in GSE84437 cohort; 51 GC patients were included in high-NRGPS group and 58 GC patients were included in the low-NRGPS group in GSE26901 cohort; 140 GC patients were included in the high-NRGPS group and 160 GC patients were included in the low-NRGPS group in GSE62254 cohort; 114 GC patients were included in the high-NRGPS group and 77 GC patients were included in the low-NRGPS group in GSE15459 cohort. The TCGA cohort’s NRGPS distribution, survival status, and survival time were comparable in the GEO cohorts (**Figures 5A–D**). The results proved that the NRGPS has excellent stability.

**Figure 6:**
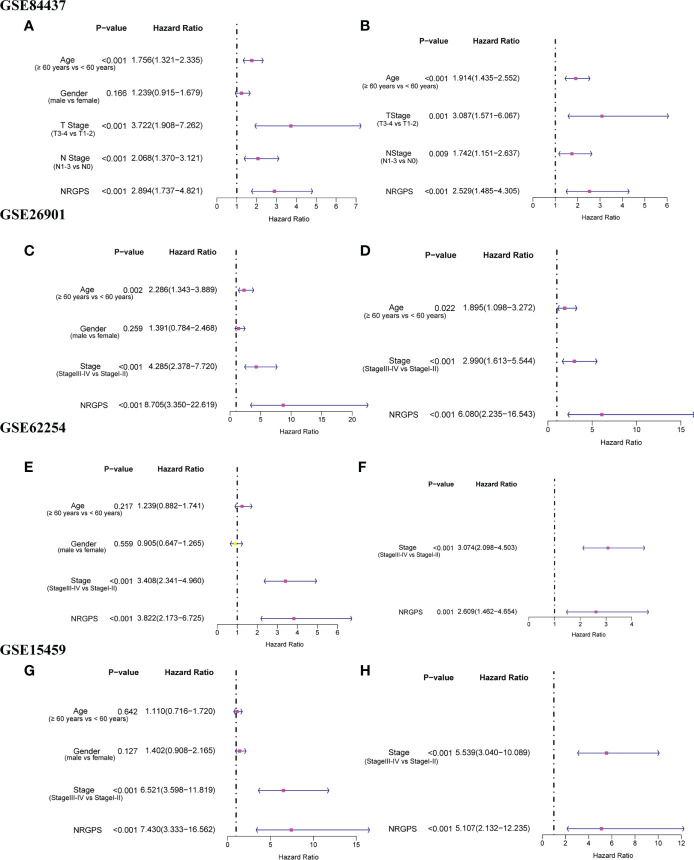
Independent prognostic analysis of prognostic model in GEO validation cohorts was performed. The forestplot of univariable Cox regression analysis of NRGPS and clinical characteristics in **(A)** GSE84437, **(C)** GSE62901, **(E)** GSE62254, **(G)** GSE15459. The forestplot of multivariable Cox regression analysis of NRGPS and clinical characteristics in **(B)** GSE84437, **(D)** GSE62901, **(F)** GSE62254, **(H)** GSE15459. NRGPS,necroptosis-related gene prognostic score.

In addition, we also analyzed Kaplan–Meier curve and the AUC in the time-dependent ROC analysis of four GEO cohorts to further verify NRGPS. As shown in **Figures 5E–H**, the OS of the high-NRGPS group was lower than of the low-NRGPS group.The AUC in the time-dependent ROC curve of the four cohorts showed that NRGPS had excellent predictive efficacy (**Figures 5I–L**). Finally, we performed univariable and multivariable Cox analyses on four GEO validation cohorts to further determine the independent predictors that NRGPS can be used (**Figures 6A–H**).

### Nomogram and GSEA analysis of NRGPS

**Figure 7:**
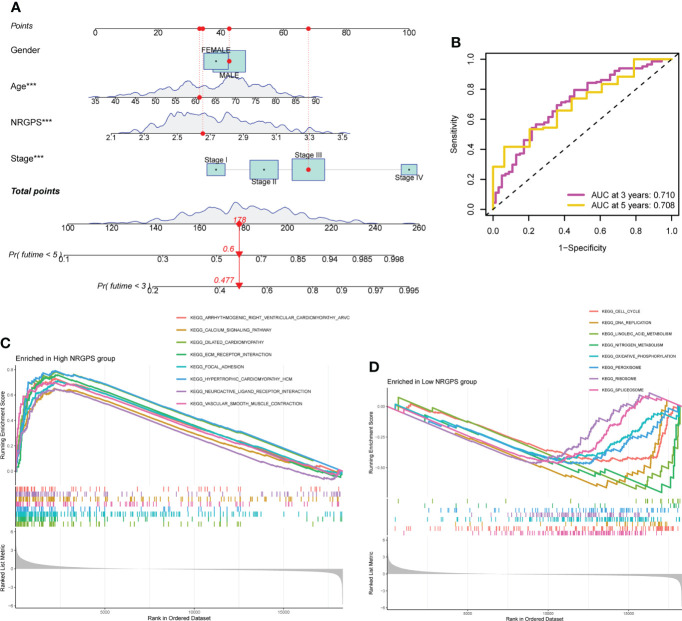
Nomograph Model and GSEA Analysis. **(A)** Nomogram of NRGPS and clinical factors predicting survival probability of GC patients. **(B)** The ROC curve verifies the predictive ability of the nomogram. **(C)** Enrichment pathways in high-NRGPS group. **(D)** Enrichment pathways in low-NRGPS group. GSEA, Gene Set Enrichment Analysis; NRGPS, necroptosis-related gene prognostic score; GC, Gastric cancer; ROC, receiver operating characteristic. ***p < 0.001.

To help in determining each patient’s specific prognosis for GC, we developed a nomogram based on NRGPS and clinical characteristics. In order to accurately evaluate the prognosis of each patient with GC, we constructed a 3-year and 5-year prognostic nomogram model combined with age, gender, stage and NRGPS (**Figure 7A**). For example, when a 60-year-old male patient is in stage III and NRGPS of 2.7, he will get 178 total points, which means that his probability of survival in less than 3 years and less than 5 years is 0.477 and 0.6, respectively. Next, we demonstrate the nomogram model’s predictive power using a time-dependent ROC analysis. The AUC is 0.710 in 3 years and 0.708 in 5 years (**Figure 7B**). At the same time, four GEO cohorts were used to verify the Nomogram model (**Figures S2**, **S3**). For GC patients in the GSE15459 cohort, a Nomogram model was used to predict the prognosis, and the AUC reached 0.872 in 3 years, 0.891 in 5 years (**Figure S3B**). These results suggest that the nomogram has accurate and stable prognostic ability.

Based on the KEGG gene sets in the high- and low-NRGPS groups, GSEA was carried out to investigate variations in biological characteristics between the two groups.The enriched pathways in the high-NRGPS group were “Neuroactive ligand-receptor interaction”, “Calcium signaling pathway”, “Vascular smooth muscle contraction”, and “Focal adhesion” (**Figure 7C**). The enriched pathways in the low-NRGPS group were “DNA replication” and “cell cycle” (**Figure 7D**). These findings demonstrated that NRGPS can reliably detect tumor progression and that the high-NRGPS group was related to tumor development and metastasis.

### Analysis of TME

**Figure 8:**
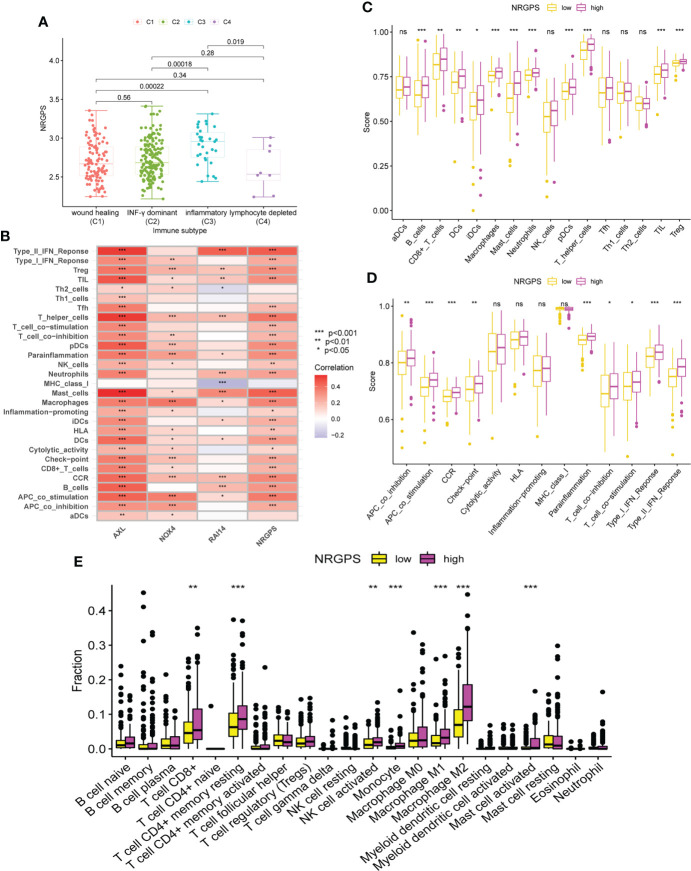
Analysis of immune subtypes and immune cell infiltration in the TCGA cohort. **(A)** The boxplot of differences in NRGPS between immune subtype groups. **(B)** Correlation analysis of NRGPS and 3 candidate genes with immune cells and signaling pathways (ssGSEA algorithm). **(C)** The boxplot of 16 immune cell differences in the low- and high-NRGPS groups (ssGSEA algorithm). **(D)** The boxplot of 13 immune signaling pathway differences in the low- and high-NRGPS groups (ssGSEA algorithm). **(E)** The boxplot of 22 immune cell infiltration differences between high-NRGPS and low-NRGPS groups in TCGA cohort (CIBERSORT-ABS algorithm). NRGPS, necroptosis-related gene prognostic score; ssGSEA, single sample gene set enrichment analysis. *p < 0.05, **p < 0.01, ***p < 0.001, ns, not significant.

By analyzing human tumors in TCGA, six kinds of immune infiltration were identified, namely wound healing (C1), INF-γ dominant (C2), inflammatory (C3), lymphocyte depleted (C4), immunologically quiet (C5), and TGF-β dominant (C6) (18), corresponding to tumor promotion and tumor inhibition, respectively. No GC patient belonged to the C5 subtype, and only four GC samples belonged to the C6 subtype; thus, C5 and C6 were not included in this study. According to our analysis of the associations between the GC sample subtypes and NRGPS, C4 was related with low-NRGPS groups, whereas C3 was connected with high-NRGPS groups (**Figure 8A**).

The activation of 13 immune-related pathways and the infiltrating status of 16 immune cells in the TCGA cohort were investigated using ssGSEA. As shown in **Figure 8B**, the three candidate genes and NRGPS are closely related to immune cells and pathways. Next, we discovered that the level of immune cell infiltration in the high-NRGPS group in TCGA cohort was higher, especially with respect to B-cells, CD8 T cells, dendritic cells, immature dendritic cells, Macrophages cells, mast cells, neutrophils, plasmacytoid dendritic cells, helper T cells, tumor-infiltrating lymphocytes, and regulatory T cells (than in the low-NRGPS group) (**Figure 8C**). Moreover, the activity of nine immune pathways, i.e., antigen-presenting cell co-inhibition, antigen-presenting cell co-stimulation, C–C chemokine receptor, Check-point, para-inflammation, T cell co-inhibition, T cell co-stimulation, type I interferon response, and type II interferon response in the high-NRGPS group was higher than that in the low-NRGPS group in TCGA cohort (**Figure 8D**). The relatively high level of immune cell infiltration and pathway activation in the high-NRGPS group was also consistent with the C3 subtype of GC tissues, with the highest number of “inflammatory” features, as shown in our previous results. In addition, We analyzed the immune cell between the high- and low-NRGPS groups in TCGA cohort using the CIBERSORT-ABS algorithm. The levels of CD8 T cells,resting memory CD4 T cells, activated NK cells, Monocytes, M1 Macrophages, M2 Macrophages, and activated Mast cells were higher in high-NRGPS group than in the low-NRGPS group (**Figure 8E**). Again, this supports the results of the above analysis. On the one hand, this result suggests that inflammatory cells are associated with the prognosis of GC patients. On the other hand, it provides a basis for the possible regulatory role of NRGPS in the TME, which may affect the prognosis of GC patients.

**Figure 9:**
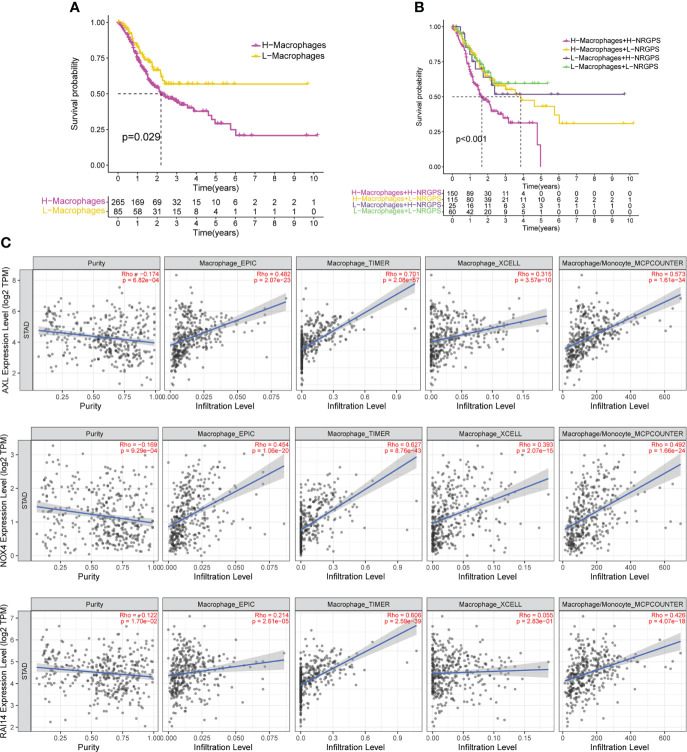
NRGPS and macrophages in TME were analyzed. **(A)** Difference in survival time between high- and low-macrophage groups (ssGSEA algorithm). **(B)** Survival analysis of macrophage combined NRGPS (ssGSEA algorithm). **(C)** EPIC, TIMER, XCELL and MCPCOUNTER algorithms were used to analyze the correlation between the three candidate genes and macrophages. NRGPS, necroptosis-related gene prognostic score; TME, tumor microenvironment.

Since differences in the degree of macrophage infiltration were observed in the high- and low-NRGPS groups, the OS of GC patients with different macrophage infiltration was analyzed. It can be found from the results that the OS of GC patients with high macrophage infiltration is relatively low (**Figure 9A**). Next, we combined macrophages and NRGPS to analyze the survival of patients. High-macrophages-high-NRGPS patients had the lowest OS (**Figure 9B**). The results confirmed our speculations. Finally, we confirmed the close relationship between the three candidate genes and macrophages through the algorithms of EPIC, TIMER, xCELL and MCPCOUNTER (**Figure 9C**).

### The effect of immunotherapy in the different NRGPS groups

**Figure 10:**
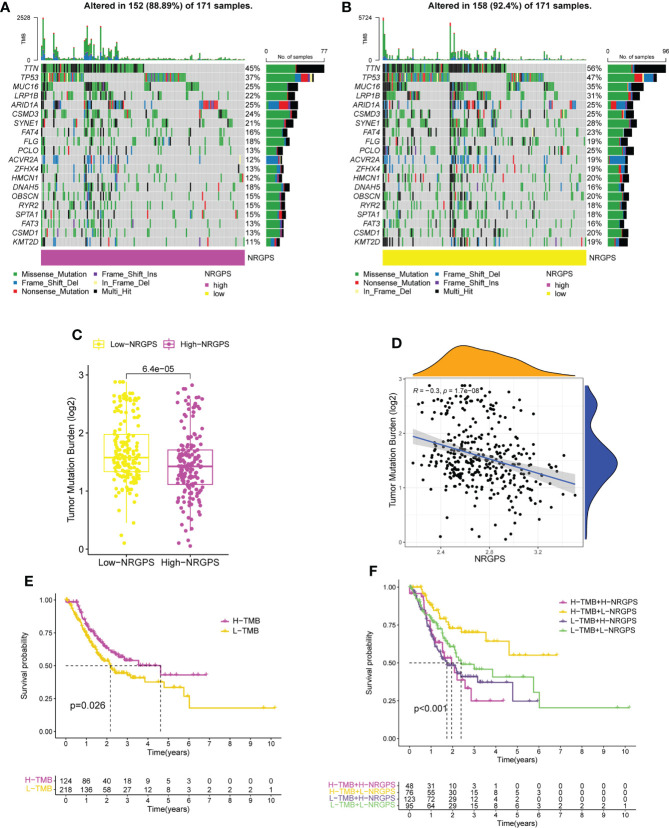
TMB analysis and survival analysis of patients in TCGA cohort. **(A)** The waterfall plot of somatic mutation in high-NRGPS group. **(B)** The waterfall plot of somatic mutation in low-NRGPS group. **(C)** The boxplot of TMB differences between low-NRGPS and high-NRGPS groups. **(D)** Correlation analysis between TMB and NRGPS. **(E)** Difference in survival time between high- and low-TMB groups. **(F)** Survival analysis of TMB combined NRGPS. TMB, Tumor mutation burden; NRGPS, necroptosis-related gene prognostic score.

To investigate the connection between somatic mutation and NRGPS in more detail, analysis was done on the variations in somatic mutation distribution between the TCGA cohort’s high-NRGPS and low-NRGPS groups. Somatic mutations were found in 88.89% of the GC patients in the high-NRGPS group and 92.4% of the GC patients in the low-NRGPS group (**Figures 10A, B**). Missense mutations, frameshift deletions, as well as nonsense mutations were the most frequent mutations in GC patients, a discovery that is in line with the findings of earlier research (19).

Additionally, the TMB of the two groups was calculated and analyzed, showing that the TMB level was significantly higher in the low-NRGPS group than in the high-NRGPS group (**Figure 10C**). The TMB was negatively correlated with NRGPS (*r* = -0.3, p< 0.001) (**Figure 10D**). The OS of the high-TMB group was higher than that of the low-TMB group (**Figure 10E**). To further prove that NRGPS and TMB could be used together for the prognostic assessment of GC, we combined the high- and low-NRGPS groups with the high- and low-TMB groups (**Figure 10F**), and the results showed that patients with high-TMB-low-NRGPS had the longest survival time, while patients with low-TMB-high-NRGPS had the shortest survival time. This indicated that the NRGPS combined with TMB level has excellent predictive power for the prognosis of GC patients.

**Figure 11:**
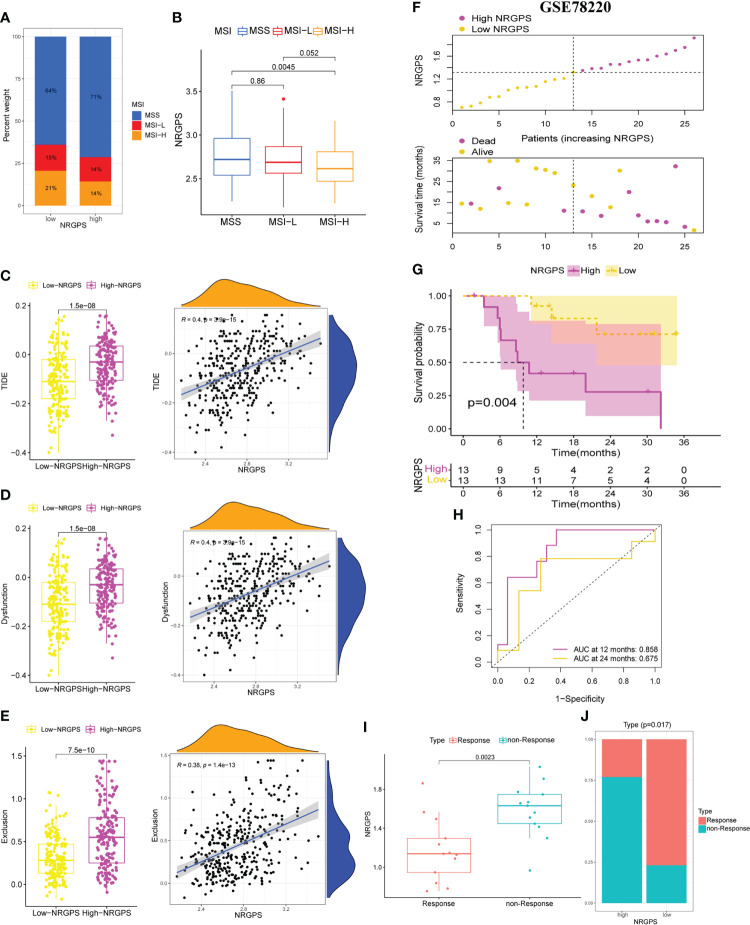
Differences in MSI statuse, TIDE score between high-NRGPS and low-NRGPS groups in the TCGA cohort. **(A)** Distribution of patients with different MSI statuses in high- and low-NRGPS groups. **(B)** The boxplot of NRGPS differences between groups with different MSI. **(C)** The boxplots of differences between TIDE in low- and high-NRGPS groups and correlation analysis of TIDE with NRGPS. **(D)** The boxplots of differences between Immune Dysfunction scores in low- and high-NRGPS groups and correlation analysis of Immune Dysfunction scores with NRGPS. **(E)** The boxplots of differences between Immune Exclusion scores in low- and high-NRGPS groups and correlation analysis of Immune Exclusion scores with NRGPS. **(F)** The distribution of NRGPS and the survival status of the high- and low-NRGPS groups. **(G)** Kaplan-meier survival analysis in GSE78220 cohort. **(H)** The ROC analysis of GSE78220 cohort. **(I)** The boxplot of NRGPS differences between immunotherapy responde and non-responde groups. **(J)** Distribution of patients who responded and did not respond to immunotherapy in the high- and low-NRGPS groups. MSI, Microsatellite Instability; TIDE, Tumor Immune Dysfunction, and Exclusion; NRGPS, necroptosis-related gene prognostic score, ROC, receiver operating characteristic.

Then, we analyzed the MSI of GC patients in the TCGA cohort. We discovered that the high-NRGPS group contained more MSS patients and fewer MSI-H patients (**Figure 11A**). This also predicted a poor prognosis for patients in the high-NRGPS group. Furthermore, patients with MSS had a higher NRGPS than those with MSI-H, demonstrating once more that NRGPS can accurately predict the prognosis of GC patients (**Figure 11B**).

In addition, we performed a correlation analysis between NRGPS and immune checkpoints. NRGPS is positively correlated with *PDCD1LG2* and *HAVCR2*, and the expression of *PDCD1LG2* and *HAVCR2* is significantly higher in GC patients in the high-NRGPS group (**Figure S4A**).

Because the TIDE score and immune escape potential are positively associated, tumor patients with higher TIDE scores are less likely to benefit from immunotherapy. Our results showed that compared with that in the high-NRGPS group, the low-NRGPS group had a lower TIDE score, and NRGPS was positively correlated with TIDE (*r* = 0.4, *p*< 0.001), implying that the low-NRGPS group GC patients might benefit more from the immunotherapy than the high-NRGPS group GC patients (**Figure 11C**). Additionally, we discovered that the T cell dysfunction and T cell exclusion scores in the high-NRGPS group were higher than those in the low-NRGPS group, and that NRGPS was also positively connected with these scores (**Figures 11D, E**). This indicates that NRGPS has a high degree of confidence in determining the effectiveness of immunotherapy.

Finally, the GSE78220 cohort verified the predictive ability of NRGPS for immunotherapy. According to the median NRGPS of the GSE78220, patients were divided into high-NRGPS group and low-NRGPS group. With the increase in NRGPS, the survival rate of patients also decreased gradually (**Figure 11F**). There was a significant difference in survival time between high- and low-NRGPS groups, and NRGPS also had good prediction ability (**Figures 11G, H**). Then, the NRGPS of the non-response group was significantly higher than that of the response group (**Figure 11I**). In addition, there are also differences in the proportion of patients with or without response to immunotherapy in the high- and low-NRGPS group, and the proportion of patients with non-response in the high-NRGPS group is larger (**Figure 11J**).

## Discussion

Necroptosis is closely related to tumorigenesis and immunity and is a target for tumor therapy. In the tumor microenvironment, necroptosis is mostly considered as pro-inflammatory cell death (20), but its underlying inflammation also has the potential to promote tumor development and metastasis through genomic instability, cell proliferation and angiogenesis proliferation and angiogenesis to promote tumor development and metastasis (21). Necroptosis plays different roles in different cancers, with necroptosis-associated protein RIPK3 being expressed at low levels in leukemia, colorectal cancer, breast cancer and melanoma relative to adjacent normal tissues, and at increased levels in lung cancer and pancreatic cancer (8). Recent research showed that necroptosis was associated with a critical role in GC development (22). However, it remains unclear whether necroptosis can predict prognosis and immunotherapy efficacy for GC patients. Typically, either TNM stage systems or serum markers (CEA, CA19-9, and CA125) are employed to monitor progression and to predict prognosis in GC patients. Nevertheless, these methods are not unsatisfactory, having low accuracy and high non-specificity, especially for GC patients with highly heterogeneous (23). In this study, a new NRGPS system for GC was established and four independent GEO external validation were performed. The ability of NRGPS to predict the effect of immunotherapy was also confirmed in the GSE78220 cohort. The results demonstrated that the NRGPS system could accurately forecast the prognosis and immunotherapy sensitivity of GC patients.

In order to understand how NRGs promote GC progression, we first obtained 35 NRGs significantly associated with GC prognosis by univariable Cox regression analysis in the TCGA cohort. Then,it was revealed by GO and KEGG pathway analysis that these NRGs were mostly implicated in several pathways linked to tumor growth.In the risk factor group, there were 5 cross-genes in TCGA and 4 independent GEO datasets. The NRGPS system for gastric cancer patients consisting of three NRGs was further constructed using lassoCox regression analysis, and this system had an excellent performance in discriminating the high-NRGPS group with a poorer prognosis. The results demonstrated that the high-NRGPS group was significantly associated with shorter survival time in TCGA and four GEO cohorts. Moreover, NRGPS could independently predict the OS for GC patients. We used the GSEA analysis to investigate the gene sets enriched in the two NRGPS groups in order to more thoroughly examine the function of these three marker genes in GC, and the results showed that the gene sets of the high-NRGPS group were mainly enriched in the “Neuroactive ligand-receptor interaction”, “Calcium signaling pathway”, “Vascular smooth muscle contraction”, and “Focal adhesion”, which are associated with tumor development (24–27).

The NRGPS was made up of three NRGs, such as *AXL*, *RAI14*, and *NOX4*. Upregulated of these genes in the GC tumor tissues was link to a poor prognosis. The tumor microenvironment’s immunosuppression and the survival, proliferation, migration, invasion, and metastasis of tumor cells are all significantly influenced by the oncogenic receptor tyrosine kinase *AXL* (28–30). *AXL* increases the expression of *ZEB1* in GC cells, promoting EMT, invasion, and proliferation (31). *AXL* inhibitors control the polarization of macrophages to boost tumor immunity (32). Targeting of *AXL* receptors is particularly well suited to enhance the efficacy of immune checkpoint inhibitors (33). Retinoic acid induced 14 (*RAI14*) was originally identified in all-trans retinoic acid-induced human retinal pigment epithelial cells (34), *RAI14* may be connected to the growth and invasion of cancer cells in several malignancies, according to current investigations (35). High expression of *RAI14* may enhance the translocation of esophageal tumor cells (36). *RAI14* is significantly expressed in GC, and its knockdown slows the development of GC (37).

The nicotinamide adenine dinucleotide phosphate (NADPH) oxidase 4 (*NOX4*) is one of the most important NADPH isoforms in endothelial cells, and it has been reported that the receptor-interacting protein 1 is involved in tumor necrosis factor-α-induced reactive oxygen species generation and necroptosis through interaction with NADPH oxidase (38). Through the recruitment of M2 TAM through the generation of different cytokines in response to ROS/PI3K signaling, tumor *NOX4* stimulates the proliferation of non-small cell lung cancer cells (39). In GC patients, the high expression of *NOX4* results in a bad prognosis (40).

We next further demonstrated that NRGPS is associated with multiple immune cell recruitment, so we further performed TME analysis.

The imbalance in the ratio of immune cell components is highly correlated with poor prognosis in cancer patients (41). Moreover, the immune cells in the TME can be used in the prognostic assessment of a variety of tumors (42). Recent studies have identified that necroptosis can regulate the TME (43). On this basis, we evaluated the GC TME based on the NRGPS. In this study, the degree of immune cell infiltration varied significantly between the groups with high and low NRGPS.In patients with high-NRGPS, the majority of immune cells were heavily invaded.

One of the most crucial elements of the tumor immunosuppressive microenvironment are tumor-associated macrophages (TAM),which is influenced by the surrounding TME, and macrophages show a continuous activation state (44, 45). In our results, TAM infiltration was relatively high in the high-NRGPS group and was abundant in immune cells recruited by NRGPS, *AXL*, *RAI14*, and *NOX4*, and in the combined TAM and NRGPS analysis results, we found that GC patients in the high-TAM and high-NRGPS groups had the shortest survival times.M1-like macrophages and M2-like macrophages are the two main classifications of activated macrophages. The inflammatory response is intimately correlated with both M1 and M2 macrophages, with M1 macrophages primarily generating pro-inflammatory cytokines to take part in the pro-inflammatory response., stimulating the Th1 response of T cells (IFN-γ) and further enhancing the M1 macrophage response, and M2 macrophages mainly participating in the anti-inflammatory response (46, 47).

It is well known that M1 macrophages promote the attack on tumor cells, while M2 macrophages have been consistently associated with cancer metastasis and poor prognosis (48), but it has also been suggested that CD68+ HLA-DR+ M1-type macrophages in the tumor microenvironment can promote tumor migration through the NF-KB signaling pathway, which in turn promotes tumor progression (49). Moreover, recent studies have shown that M1-type macrophages polarized by exosomes promote the malignant migration of oral squamous cell carcinoma (50). It is clear that the complexity of the tumor microenvironment is not only in the differences between individuals with different diseases, but also in the different parts of the same tumor tissues of patients, which may have different effects depending on the microenvironment in which the tumor is located (51–53).

Thus, It is obvious that the biology of M1-type macrophages in relation to cancer is complex and fascinating.Additionally, we discovered that patients with GC in the high-NRGPS group with pro-tumor immune cell infiltration had a much shorter survival time than those in the low-NRGPS group. This suggests that in the high-NRGPS group, these immunosuppressive cells were manipulated to protect them from the body’s immune response, which is also known as “sabotage” (54).

Ultimately, the tuRecent studies have demonstrated the significant association of TMB and TME in GC patients regarding tumorigenesis, tumor progression, and drug resistance (55). Nevertheless, the exact mechanisms by which necroptosis plays a role during tumor immunotherapy remain largely unknowable. We further analyzed the TMB and TIDE in the different subgroups to assess the immunotherapy response.

The TMB is another index to evaluate patient reaction to immunotherapy independent of the programmed cell death-ligand 1 expression level (56, 57). A comprehensive analysis of 27 cancer types showed a positive correlation of benefit between TMB and immunotherapy (58). According to a recent Panel Sequencing study, patients with elevated TMB had longer OS., and tests for treatment response to immunotherapy were performed, suggesting that TMB could be used as a predictive biomarker for advanced GC patients treated with immunotherapy (59). In the field of cancer biomarkers, although thousands of expression signatures have been nominated to be used as biomarkers, few have found reliable clinical use, therefore the expression signature of the marker must be consistent with reproducible genetic marker alterations (60).

Consistently, in our study, the TMB was lower in the high-NRGPS group than in the low-NRGPS group. The NRGPS was negatively correlated with the TMB, and the combination of NRGPS with the TMB interestingly revealed that GC patients in the high-TMB and low-NRGPS groups had the longest survival time, and GC patients in the low-TMB and high-NRGPS groups had the shortest survival time, demonstrating the reliability of NRGPS as a prognostic evaluation index for the immunotherapy of GC patients. In China, about 95% of GC patients have the characteristics of microsatellite stability (MSS) (61). Patients with GC in the MSI-H group had a higher survival rate and significantly more benefit from immunotherapy in contrast to the MSS/MSI-L group (62, 63). Consistently, according to our research, the high-NRGPS group of GC patients had a larger percentage of MSS and a lower percentage of MSI-H.

Programmed cell death-ligand 1 level and TMB are less accurate than the TIDE at predicting survival outcomes in cancer patients receiving immunotherapy medications (64, 65). Recent research has demonstrated its usefulness in predicting or evaluating the impact of immunotherapy (66–68), and it has also been affirmed in GC studies (69). Because anti-tumor immune escape is more likely in patients with higher TIDE scores, immunotherapy response rates are lower in these patients (64). Consistent with the TMB result, the GC patients with more immune dysfunction in the high-NRGPS group were more likely to resort to immunotherapy and receive immunotherapy less effectively than those in the low-NRGPS group.mor escapes immune surveillance and flees the immune system.

Our findings show that the high-NRGPS group was characterized by a pro-cancer immune microenvironment, high TIDE score, and low TMB, low MSI, which demonstrated that the high-NRGPS group was correlated with immune escape in GC, and therefore NRGPS could be used as a new biomarker to accurately predict the prognosis and immunotherapy efficacy in GC patients. Some limitations must be highlighted. First, A large number of clinical specimens and relevant information should be used to further verify the prognostic characteristics of NRGs. Second, the mechanisms by which necroptosis shapes the TME features in GC are unclear and would be experimentally investigated in the future.

## Conclusion

In conclusion, our study defined a new prognostic signal composed of three necroptosis-related genes and validated it using RT-qPCR methodsas, well as independent external validation using the GSE84437, GSE26901, GSE62254 and GSE15459 datasets. This signal was verified in GC prognosis and immunotherapy effects and was proven to be independently related with OS in the TCGA cohort and the GEO validation cohorts.It is a prognostic classifier that can be applied to clinical decision-making for therapy and personalised prognosis.

## Data availability statement

The original contributions presented in the study are included in the article/**Supplementary Material**. Further inquiries can be directed to the corresponding authors.

## Ethics statement

The studies involving human participants were reviewed and approved by The Second Affiliated Hospital of Harbin Medical University. The patients/participants provided their written informed consent to participate in this study.

## Author contributions

YX, RZZ performed the experiments. YX, RZZ, and JQL wrote and revised the manuscript. XMJ, JMD, and MZW helped or performed experiments and analyses. JQR, TG and KTH downloaded and collated the data. YHS and SYZ was responsible for supervising the study. All authors read and gave final approval ofthe manuscript.

## Funding

The present study was supported by the National Natural Science Foundation of China (No. 81772253); the Natural Science Foundation of Heilongjiang Province (No.LH2021H082); Scientific research project of Heilongjiang Provincial Health Commission (No. 2017-085).

## Acknowledgments

**Figure 1** was produced with the assistance of Servier Medical Art (https://smart.servier.com).

## Conflict of interest

The authors declare that the research was conducted in the absence of any commercial or financial relationships that could be construed as a potential conflict of interest.

## Publisher’s note

All claims expressed in this article are solely those of the authors and do not necessarily represent those of their affiliated organizations, or those of the publisher, the editors and the reviewers. Any product that may be evaluated in this article, or claim that may be made by its manufacturer, is not guaranteed or endorsed by the publisher.
