# Supplementary material for: Development and validation of a necroptosis-related gene prognostic score to predict prognosis and efficiency of immunotherapy in gastric cancer
Source: Front Immunol. 2022 Aug 26;13:977338. doi: 10.3389/fimmu.2022.977338 (PMC9504871; doi:10.3389/fimmu.2022.977338)
Supplement: Supplementary file 1 [file DataSheet_1.docx]

Supplementary Material

# Supplementary Figure S1. Stratification analyses by age, gender, and pathologic stage in all patients with GC.


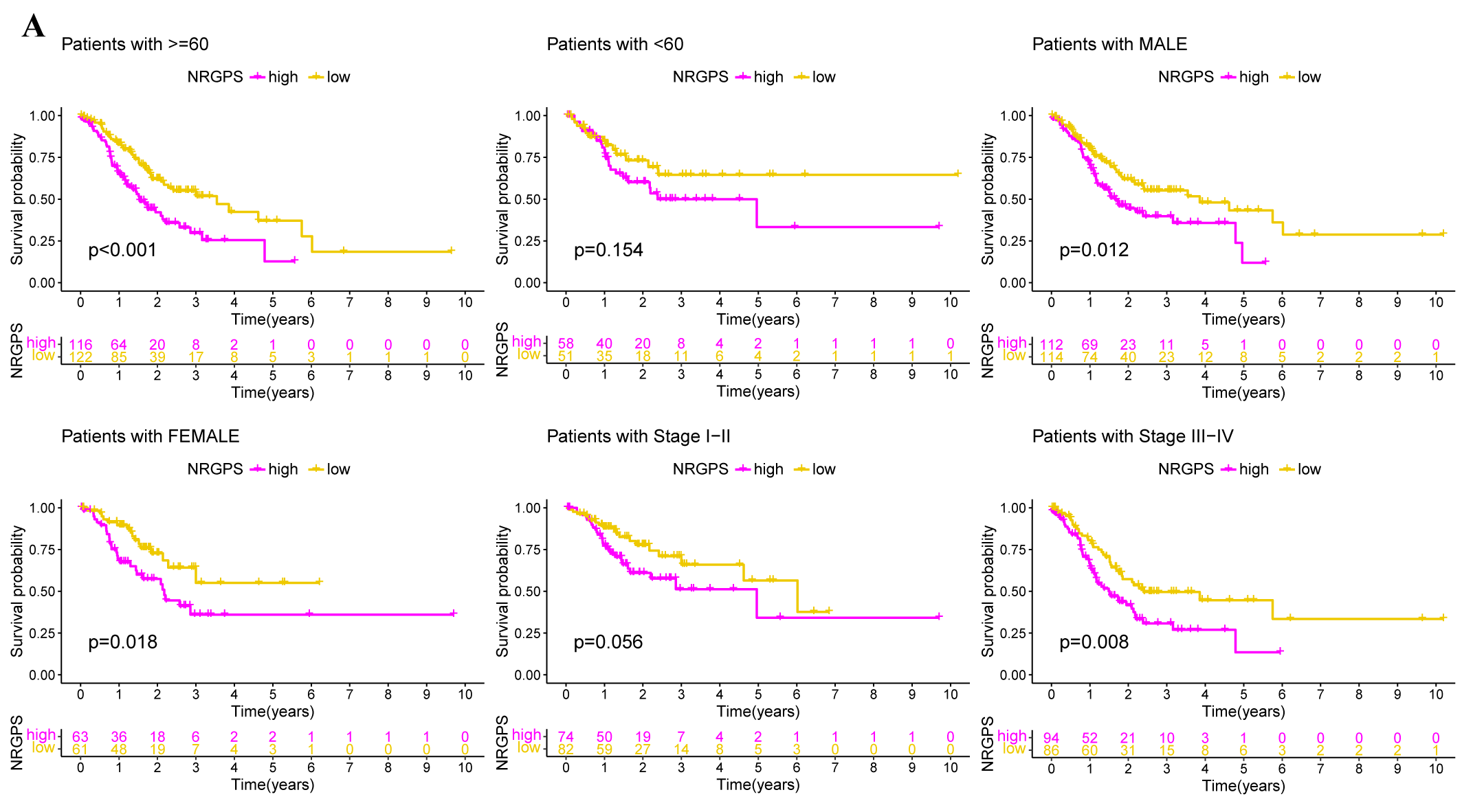


FIGURE S1 Stratification analyses by age, gender, and pathologic stage in all patients with GC.

1. Kaplan-meier survival analysis of OS prognostic value stratified by clinicopathologic characteristics.

GC, Gastric cancer; OS, Overall survival.

# Supplementary Figure S2. Validation of the nomogram model.


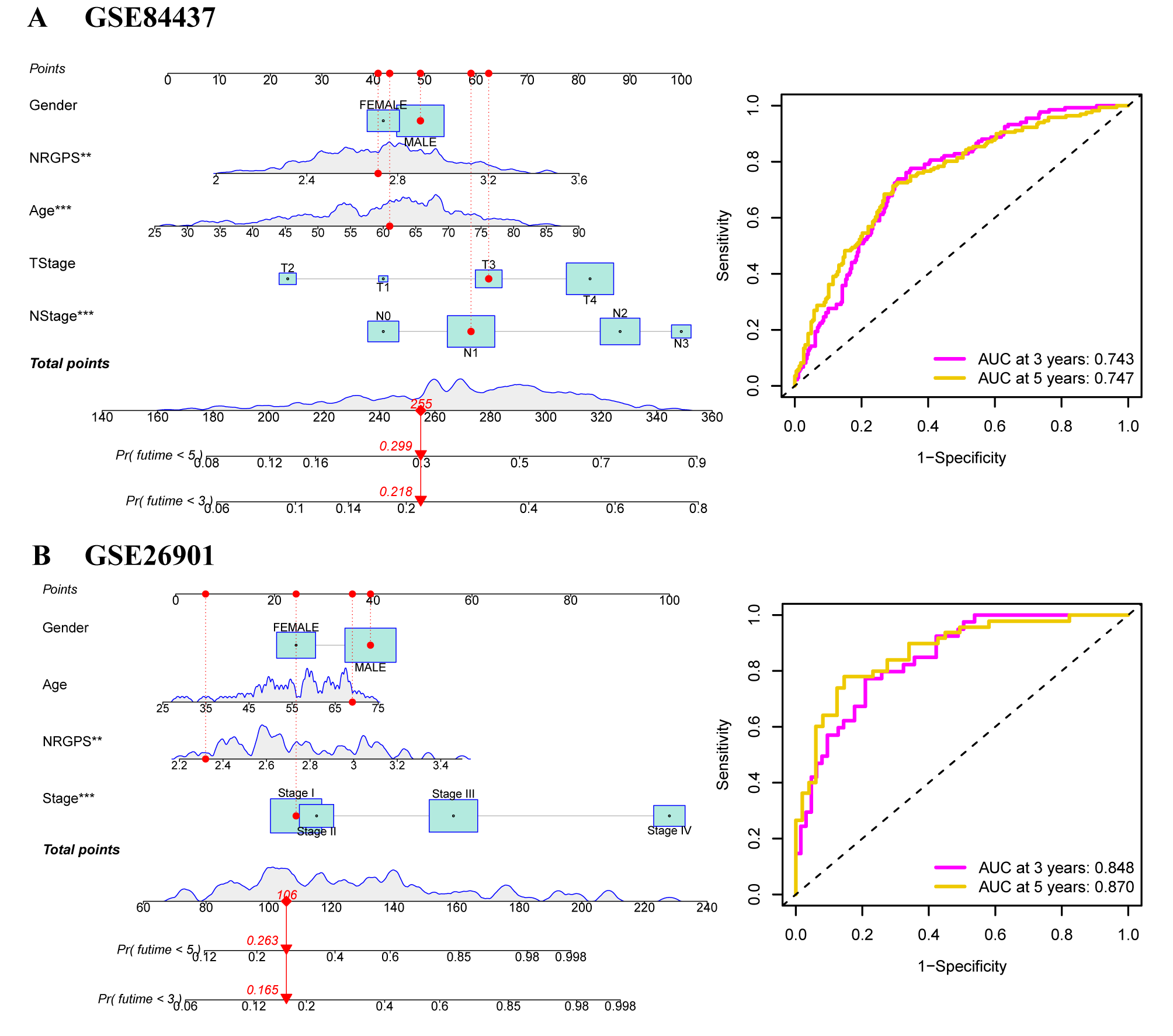


FIGURE S2 Validation of the nomogram model.

1. Nomogram of NRGPS and clinical factors predicting survival probability of GC patients in GSE84437. The ROC curve verifies the predictive ability of the nomogram.
2. Nomogram of NRGPS and clinical factors predicting survival probability of GC patients in GSE26901. The ROC curve verifies the predictive ability of the nomogram.

NRGPS, necroptosis-related gene prognostic score; GC, Gastric cancer; ROC, receiver operating characteristic.

# Supplementary Figure S3. Validation of the nomogram model.
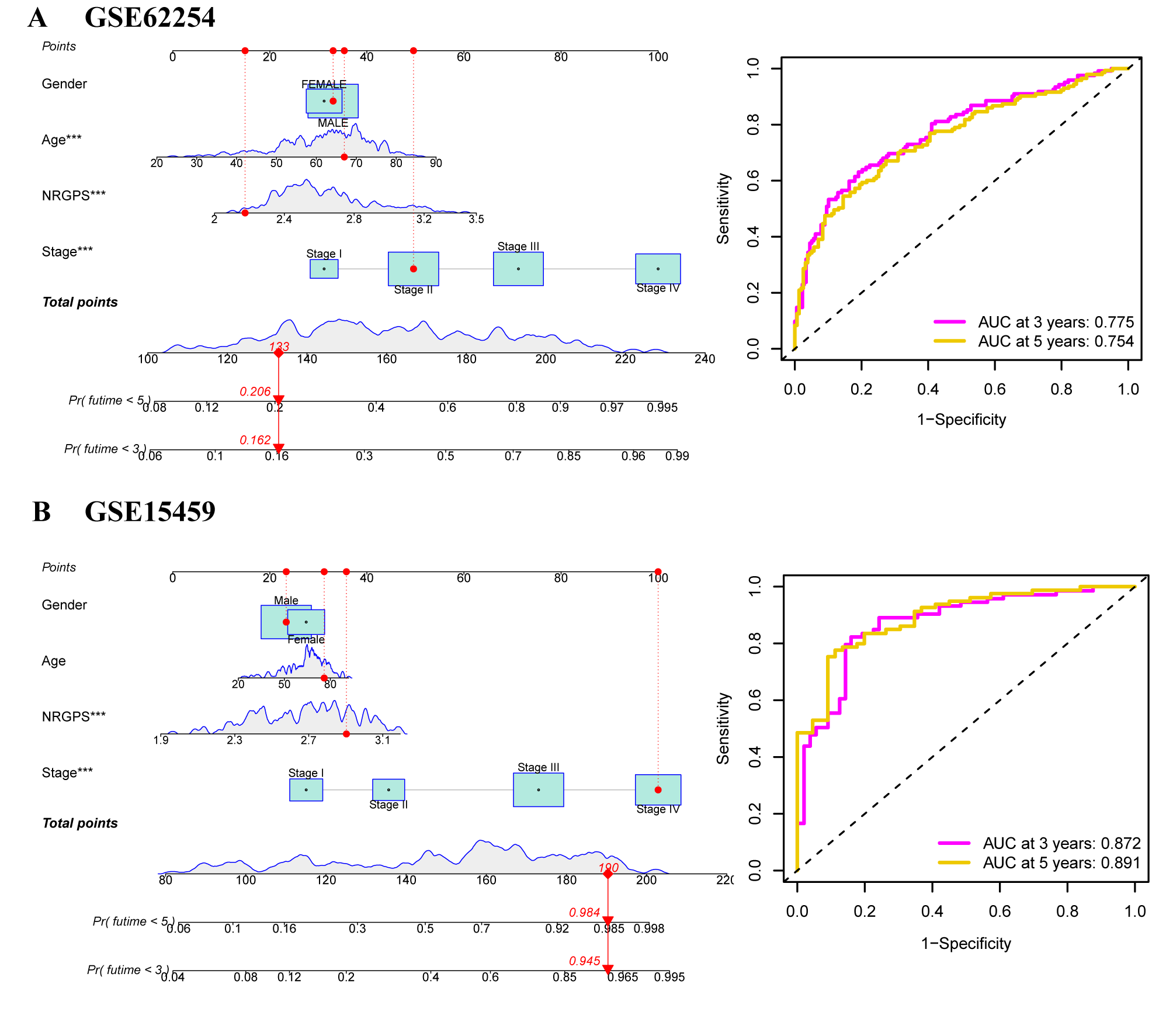


FIGURE S3 Validation of the nomogram model.

1. Nomogram of NRGPS and clinical factors predicting survival probability of GC patients in GSE62254. The ROC curve verifies the predictive ability of the nomogram.
2. Nomogram of NRGPS and clinical factors predicting survival probability of GC patients in GSE15459. The ROC curve verifies the predictive ability of the nomogram.

NRGPS, necroptosis-related gene prognostic score; GC, Gastric cancer; ROC, receiver operating characteristic.

# Supplementary Figure S4. Correlation analysis between NRGPS and immune checkpoint.
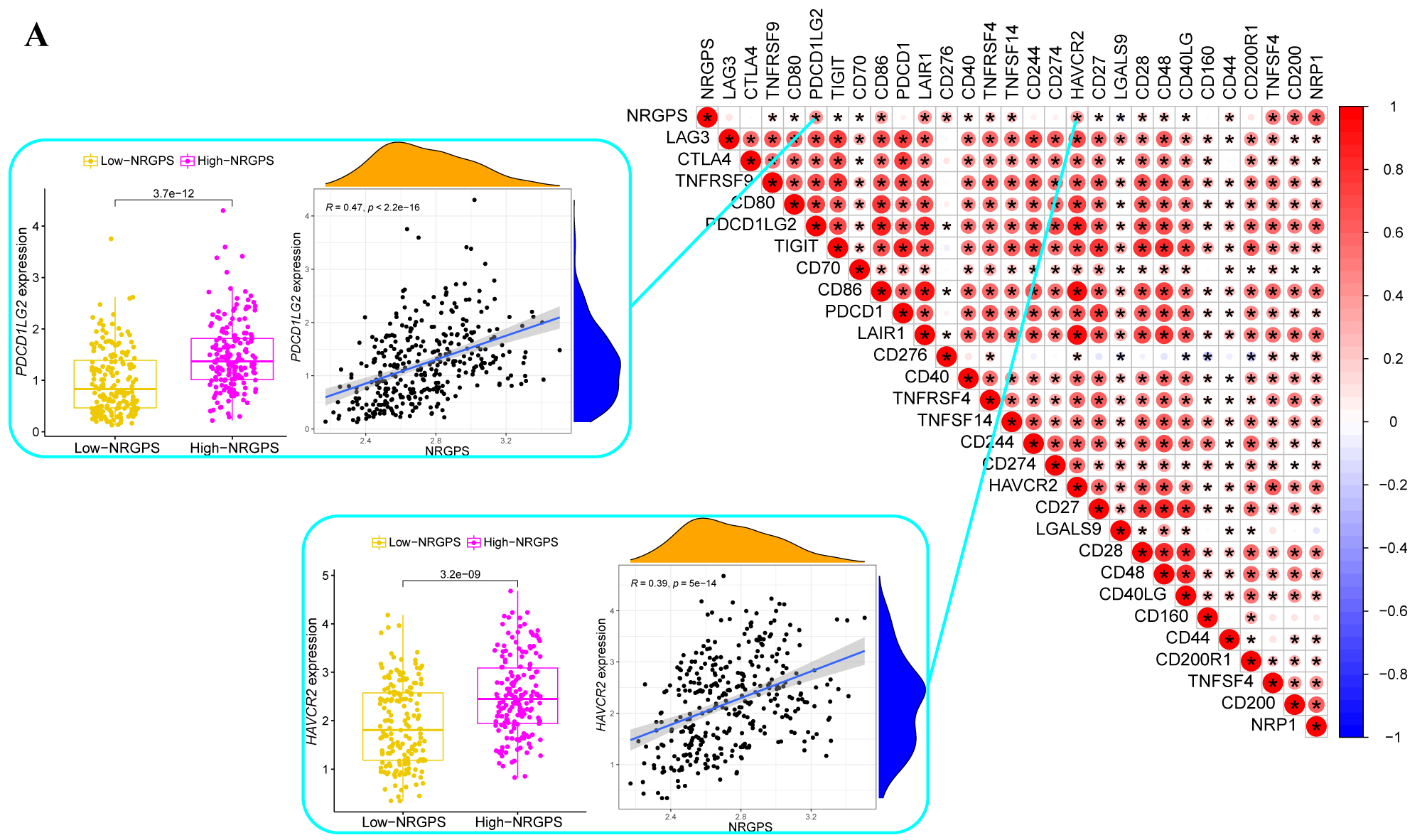


FIGURE S4 Correlation analysis between NRGPS and immune checkpoint.

1. The correlation plot between NRGPS and immune checkpoint and the difference in immune checkpoint expression between high- and low-NRGPS groups.

NRGPS, necroptosis-related gene prognostic score.

# Supplementary Table S1. Clinicopathological data.

| Pathological typing | n |
| --- | --- |
| Gastric adenocarcinoma | 31 |
| Grade |  |
| Low- differentiation | 22 |
| Moderate- differentiation | 6 |
| Well- differentiation | 3 |
| Stage |  |
| Stage I | 4 |
| Stage II | 10 |
| Stage III | 13 |
| Stage IV | 4 |

# Supplementary Table S2. Primers used for RT-qPCR.

| **Gene** | **Primer nucleotide sequence** |
| --- | --- |
| *AXL* | Forward: TTTCCTGAGTGAAGCGGTCT |
|  | Reverse: GAGGAGGAAGCTGTGTAGGT |
| *RAI14* | Forward: AGGGCAGAACTGGTATGCTT |
|  | Reverse: CAGGTGAGACAAGACCGAGT |
| *NOX4* | Forward: AACCGAACCAGCTCTCAGAA |
|  | Reverse: AGCTTGGAATCTGGGCTCTT |
| *GAPDH* | Forward: TGCACCACCAACTGCTTAGC |
|  | Reverse: GGCATGGACTGTGGTCATGAG |

# Supplementary Table S3. Four GEO cohorts prognostic markers.

| GSE84437 | | | |
| --- | --- | --- | --- |
| Symbol | *p*-Value | HR-Value | 95%CI |
| *ZSCAN20* | 0.0031487 | 0.465176305 | 0.27989591-0.77310525 |
| *CPSF3* | 0.000477231 | 0.494379922 | 0.33297035-0.73403384 |
| *TRAF3* | 0.00031319 | 0.516001555 | 0.36007084-0.73945895 |
| *FASLG* | 0.0001259 | 0.520342433 | 0.3726212-0.72662598 |
| *HSPA9* | 0.000251759 | 0.524062433 | 0.37079091-0.74069084 |
| *FUS* | 0.001531954 | 0.545620783 | 0.37509441-0.79367228 |
| *RPS14* | 0.006879217 | 0.560133485 | 0.36791828-0.8527696 |
| *TRAF6* | 0.029300334 | 0.561947575 | 0.33465407-0.94361642 |
| *AIFM1* | 0.001402402 | 0.564998974 | 0.39801716-0.80203537 |
| *TCOF1* | 0.021857826 | 0.565354284 | 0.34721513-0.92054014 |
| *CCT8* | 0.004467565 | 0.56843865 | 0.38509603-0.83906994 |
| *MAPK1* | 0.013225131 | 0.575337809 | 0.37154207-0.89091821 |
| *CPSF2* | 0.016056381 | 0.576890948 | 0.3686455-0.9027729 |
| *RPS6* | 0.009073964 | 0.594336371 | 0.4020636-0.87855684 |
| *DNAJA2* | 0.017152597 | 0.615763284 | 0.41327764-0.91745691 |
| *CASP8* | 0.040978191 | 0.62045103 | 0.39256655-0.98062223 |
| *MLKL* | 0.001577087 | 0.62316613 | 0.46474322-0.83559267 |
| *ZNF24* | 0.016329635 | 0.626087618 | 0.42722638-0.91751288 |
| *SERBP1* | 0.01981305 | 0.626638719 | 0.42292303-0.92848119 |
| *ZC3HAV1* | 0.030692368 | 0.631599683 | 0.41634346-0.95814681 |
| *WDR77* | 0.007210154 | 0.632408074 | 0.45272957-0.88339706 |
| *BECN1* | 0.014984965 | 0.633303349 | 0.43830685-0.91505101 |
| *GSDMD* | 0.001858953 | 0.636463013 | 0.47883224-0.84598557 |
| *HNRNPA1* | 0.011780669 | 0.637872355 | 0.44955337-0.90507861 |
| *KRT2* | 0.013537999 | 0.650605631 | 0.46253192-0.91515346 |
| *TICAM1* | 0.001369702 | 0.653702153 | 0.50389045-0.84805438 |
| *DNAJA1* | 0.012883904 | 0.656454365 | 0.47113379-0.91467083 |
| *PARK7* | 0.030415561 | 0.659504366 | 0.45240647-0.96140538 |
| *HNRNPF* | 0.0311366 | 0.662352448 | 0.45540184-0.96334868 |
| *CASP10* | 0.022549126 | 0.663414956 | 0.46628594-0.94388307 |
| *TRAFD1* | 0.016835451 | 0.665298985 | 0.47631653-0.92926177 |
| *TUBA1B* | 0.034928825 | 0.67108445 | 0.46324354-0.9721762 |
| *ELAVL1* | 0.035750479 | 0.675801125 | 0.46877835-0.97424969 |
| *RFWD3* | 0.004494562 | 0.676709264 | 0.51690037-0.8859259 |
| *CLEC7A* | 0.043950641 | 0.706262081 | 0.5035296-0.99061928 |
| *EZH2* | 0.005462567 | 0.714129492 | 0.56315425-0.90557948 |
| *IKBKE* | 0.012032011 | 0.71487075 | 0.55011553-0.92896885 |
| *TNFRSF10B* | 0.01308683 | 0.716116497 | 0.55009876-0.93223777 |
| *TRIM24* | 0.007165163 | 0.716440998 | 0.56185822-0.91355378 |
| *SNRPF* | 0.01115966 | 0.716505946 | 0.55385827-0.92691722 |
| *CDC7* | 0.008597511 | 0.718315853 | 0.56123074-0.91936815 |
| *RBCK1* | 0.03570211 | 0.732411104 | 0.54770508-0.97940671 |
| *FAS* | 0.00907098 | 0.733379208 | 0.58099632-0.92572886 |
| *MYO6* | 0.017410818 | 0.740005977 | 0.57736994-0.94845403 |
| *RPL34* | 0.047025294 | 0.748230787 | 0.561988-0.99619443 |
| *RBM25* | 0.048465167 | 0.756097439 | 0.57276188-0.99811694 |
| *TP53* | 0.01637941 | 0.777418096 | 0.63295173-0.95485779 |
| *GNLY* | 0.004403023 | 0.78191572 | 0.66012645-0.92617435 |
| *KIF11* | 0.016896788 | 0.789371114 | 0.6501411-0.95841772 |
| *CASP1* | 0.002246244 | 0.796488895 | 0.68832369-0.9216515 |
| *CTSS* | 0.024386109 | 0.808044766 | 0.67117901-0.97281997 |
| *XBP1* | 0.044363515 | 0.822445749 | 0.67975771-0.99508545 |
| *UHRF1* | 0.049674295 | 0.827113013 | 0.68430081-0.99972983 |
| *MYC* | 0.037369998 | 0.833069318 | 0.70145648-0.9893764 |
| *BIRC3* | 0.042077228 | 0.854609026 | 0.73447718-0.99438976 |
| *KRT6A* | 0.015081944 | 1.100268534 | 1.01866807-1.18840561 |
| *C7* | 0.001166273 | 1.129380634 | 1.04940884-1.2154468 |
| *KRT5* | 0.009780829 | 1.145157411 | 1.03325379-1.26918043 |
| *UCHL1* | 0.014071058 | 1.162321637 | 1.03081824-1.31060116 |
| *KRT16* | 0.001852452 | 1.169352008 | 1.05965729-1.29040222 |
| *FLNC* | 0.000221457 | 1.174582795 | 1.0784416-1.27929481 |
| *S100A4* | 0.016768826 | 1.179945143 | 1.03032209-1.35129641 |
| *BNIP3* | 0.017349556 | 1.189923217 | 1.03111592-1.37318921 |
| *FAM83D* | 0.008827182 | 1.202017134 | 1.04737218-1.37949549 |
| *KRT7* | 5.75E-06 | 1.218908353 | 1.11896708-1.32777595 |
| *ACTC1* | 0.002787588 | 1.224424854 | 1.07225154-1.39819452 |
| *KRT14* | 2.12E-06 | 1.225944247 | 1.12694122-1.3336448 |
| *ACHE* | 0.008674074 | 1.226342335 | 1.05302924-1.42818021 |
| *AXL* | 0.019805966 | 1.238292056 | 1.03452669-1.48219203 |
| *MAP1LC3A* | 0.035412148 | 1.238815738 | 1.01473155-1.51238466 |
| *PPP1R12C* | 0.036123868 | 1.240685064 | 1.01404974-1.51797231 |
| *MIB2* | 0.026846937 | 1.241059268 | 1.02506623-1.50256448 |
| *LEF1* | 0.024274842 | 1.241079932 | 1.02846859-1.49764359 |
| *FAP* | 0.002773828 | 1.249111718 | 1.07973102-1.44506368 |
| *PRKAA2* | 0.0210976 | 1.257907949 | 1.03505106-1.52874816 |
| *ANXA1* | 0.001734209 | 1.276255488 | 1.09559588-1.48670518 |
| *MAP1B* | 0.000155988 | 1.28084775 | 1.12662346-1.45618391 |
| *MYO1D* | 0.027505451 | 1.286544168 | 1.0283189-1.60961341 |
| *LITAF* | 0.035500576 | 1.286892899 | 1.01725826-1.62799694 |
| *TP63* | 0.004005272 | 1.301832671 | 1.08776327-1.55803046 |
| *SOX17* | 0.020353033 | 1.304603631 | 1.04210489-1.63322391 |
| *TPM2* | 9.44E-07 | 1.309369967 | 1.1756212-1.45833515 |
| *TRIP6* | 0.004361589 | 1.315750673 | 1.08952891-1.58894346 |
| *TP53I3* | 0.047295153 | 1.320655286 | 1.00332619-1.7383483 |
| *SVIL* | 4.82E-05 | 1.329499422 | 1.15888373-1.52523387 |
| *KL* | 0.005719455 | 1.332694593 | 1.08709097-1.6337868 |
| *TNFRSF21* | 0.003073387 | 1.337703076 | 1.10331402-1.62188596 |
| *TPM1* | 3.33E-05 | 1.345064722 | 1.16932897-1.54721139 |
| *LGALS1* | 0.000266713 | 1.346012861 | 1.14728209-1.57916752 |
| *GSN* | 0.000453997 | 1.34605871 | 1.14005026-1.58929314 |
| *PINK1* | 0.024131491 | 1.363618046 | 1.04141596-1.78550574 |
| *NOX4* | 0.001234687 | 1.37151837 | 1.1323139-1.66125545 |
| *TPM4* | 0.006086743 | 1.413632846 | 1.10387259-1.81031565 |
| *TUBB6* | 7.17E-06 | 1.415951292 | 1.21643822-1.64818733 |
| *MYL6* | 0.03610111 | 1.469062658 | 1.02523984-2.10501486 |
| *CALU* | 0.00361632 | 1.475257114 | 1.13533456-1.91695349 |
| *FLII* | 0.013818872 | 1.528213754 | 1.09032-2.14197417 |
| *MYO1C* | 0.001109489 | 1.557632231 | 1.19342154-2.03299344 |
| *RAI14* | 3.88E-06 | 1.593926191 | 1.30777678-1.9426868 |
| *FLOT1* | 9.17E-05 | 1.771753459 | 1.33025285-2.35978471 |

| GSE62254 | | | |
| --- | --- | --- | --- |
| Symbol | *p*-Value | HR-Value | 95%CI |
| *TMOD3* | 4.53E-05 | 0.370637633 | 0.230042474-0.597160397 |
| *CASP10* | 5.61E-08 | 0.393292004 | 0.28083566-0.550779772 |
| *DIABLO* | 0.024586532 | 0.418780219 | 0.19605748-0.894517623 |
| *IPMK* | 0.000124622 | 0.455891583 | 0.305210282-0.680963739 |
| *YWHAE* | 1.22E-06 | 0.459868577 | 0.336034564-0.629337368 |
| *LRRC59* | 0.000230142 | 0.469354923 | 0.313837595-0.70193644 |
| *G3BP2* | 0.000509978 | 0.471623169 | 0.308688995-0.720558287 |
| *ZAN* | 0.019264284 | 0.490295915 | 0.269917184-0.890606815 |
| *TPM3* | 0.000773568 | 0.491888058 | 0.32526422-0.743868667 |
| *CASP2* | 0.001836155 | 0.497360825 | 0.320515569-0.771780886 |
| *DNAJA1* | 0.000508432 | 0.506295919 | 0.344940501-0.743129779 |
| *PGAM5* | 6.51E-05 | 0.508616038 | 0.364995879-0.708748479 |
| *HSPD1* | 0.000552629 | 0.516699467 | 0.355229397-0.751566006 |
| *TRAFD1* | 0.00300968 | 0.522221005 | 0.339982446-0.802143703 |
| *CPSF3* | 0.011024862 | 0.530886846 | 0.325808257-0.865051261 |
| *RBM14* | 0.003810089 | 0.533577769 | 0.348665041-0.81655802 |
| *HNRNPF* | 0.001486454 | 0.535021741 | 0.363759362-0.786916555 |
| *TWF1* | 0.001050598 | 0.545951421 | 0.380126443-0.784115286 |
| *CPSF2* | 0.018598737 | 0.552391871 | 0.336970691-0.905529138 |
| *CHUK* | 0.010430242 | 0.552835105 | 0.351251838-0.870106916 |
| *SERBP1* | 0.016336147 | 0.557800022 | 0.346380477-0.898263284 |
| *TET2* | 0.043747822 | 0.559893233 | 0.318619695-0.983870231 |
| *SLC30A9* | 0.005125538 | 0.560192454 | 0.373349794-0.840540401 |
| *RIPK1* | 0.013149624 | 0.562126498 | 0.356534262-0.886271626 |
| *OTUD4* | 0.020423622 | 0.567320616 | 0.351336287-0.916081525 |
| *IKBKE* | 0.000858674 | 0.569287169 | 0.408753197-0.792869346 |
| *HSPA5* | 0.002600188 | 0.573529871 | 0.399405795-0.8235647 |
| *RNGTT* | 0.010253685 | 0.578064329 | 0.380408677-0.878419416 |
| *PAK1* | 0.005862036 | 0.579005717 | 0.392531987-0.854064462 |
| *KIF11* | 4.68E-08 | 0.579076267 | 0.476008366-0.704460985 |
| *PPM1B* | 0.019653786 | 0.580673262 | 0.367790698-0.916775325 |
| *NPM1* | 0.031132702 | 0.580855225 | 0.354426075-0.951941226 |
| *CASP8* | 0.015901002 | 0.582017706 | 0.37485304-0.903673102 |
| *TCOF1* | 0.033429853 | 0.584075548 | 0.35584891-0.958677225 |
| *GSDMD* | 0.001621875 | 0.584357843 | 0.418401745-0.816139255 |
| *EZH2* | 3.22E-05 | 0.586347682 | 0.455881481-0.754151283 |
| *KCTD5* | 0.003219127 | 0.590749059 | 0.416215477-0.838470624 |
| *LACTB* | 0.00609815 | 0.592874973 | 0.408042722-0.861431206 |
| *MRPS12* | 0.002737632 | 0.594300932 | 0.422815532-0.835337331 |
| *NSUN2* | 0.020883204 | 0.60843087 | 0.399142707-0.927458066 |
| *CCT6A* | 0.010908142 | 0.612747725 | 0.420245219-0.893430211 |
| *WRNIP1* | 0.024923462 | 0.614422964 | 0.40141478-0.940462578 |
| *SNRPF* | 0.001583756 | 0.616127217 | 0.456214427-0.832092816 |
| *RNF31* | 0.011740295 | 0.616976817 | 0.423777866-0.898254539 |
| *HSP90AA1* | 0.014970031 | 0.621600406 | 0.423814884-0.911688283 |
| *FASLG* | 0.018321217 | 0.622560048 | 0.419935095-0.922954565 |
| *TNFRSF10A* | 0.000347266 | 0.624897515 | 0.482983388-0.80851001 |
| *PKP2* | 1.32E-06 | 0.628203277 | 0.520350055-0.758411291 |
| *PLK1* | 0.000378221 | 0.628601189 | 0.486641266-0.811972765 |
| *GCC2* | 0.044652288 | 0.635272573 | 0.407967676-0.989223573 |
| *VCP* | 0.044289592 | 0.639028599 | 0.41305529-0.988626852 |
| *MYO6* | 0.001433622 | 0.639294291 | 0.485555778-0.841710076 |
| *RARG* | 0.016885612 | 0.640339633 | 0.444221656-0.923041099 |
| *PKP3* | 0.000106236 | 0.647593282 | 0.519854092-0.806720704 |
| *UHRF1* | 8.04E-05 | 0.660467433 | 0.537411209-0.811701026 |
| *PAWR* | 0.010352844 | 0.661056838 | 0.48174421-0.907112393 |
| *TXN* | 0.008991862 | 0.67141094 | 0.49794793-0.905300782 |
| *IFNB1* | 0.038135917 | 0.6729226 | 0.462744821-0.978562709 |
| *RPS27L* | 0.021086843 | 0.67380898 | 0.48175929-0.942417824 |
| *HNRNPL* | 0.015894571 | 0.674223922 | 0.489388261-0.928869639 |
| *CALML5* | 0.032504326 | 0.674381354 | 0.469969581-0.967701378 |
| *POF1B* | 1.14E-05 | 0.684723114 | 0.578160603-0.810926481 |
| *MLKL* | 0.0345146 | 0.685858659 | 0.483506738-0.972896679 |
| *EIF4EBP1* | 0.010254229 | 0.685982867 | 0.514447775-0.914713827 |
| *PTGES3* | 0.021112753 | 0.686201513 | 0.498245002-0.945062198 |
| *CDC7* | 0.009184923 | 0.689493421 | 0.521251005-0.912038872 |
| *RFWD3* | 0.023604662 | 0.690168482 | 0.500618198-0.95148865 |
| *CTSS* | 0.000528838 | 0.693496297 | 0.563832013-0.852979439 |
| *BUB1B* | 0.001649144 | 0.694219078 | 0.5530727-0.87138658 |
| *CASP6* | 0.012394862 | 0.696355757 | 0.52438166-0.924729787 |
| *SP6* | 0.004303497 | 0.69649567 | 0.543353012-0.892801196 |
| *CASP1* | 4.54E-05 | 0.699597427 | 0.589224653-0.830645081 |
| *TICAM1* | 0.025500473 | 0.700361983 | 0.512392472-0.957287496 |
| *MYH14* | 0.000664726 | 0.712788459 | 0.586531355-0.866223746 |
| *ITPK1* | 0.03041321 | 0.714525121 | 0.527035556-0.968712914 |
| *CDC42BPG* | 0.041731088 | 0.717540985 | 0.521302756-0.987650763 |
| *IDH2* | 0.006320667 | 0.72742425 | 0.578874575-0.914094455 |
| *TNFRSF10B* | 0.045064957 | 0.731579424 | 0.538900822-0.993148333 |
| *ZBP1* | 0.015716119 | 0.733265477 | 0.570072468-0.94317528 |
| *RIPK3* | 0.030013807 | 0.735998535 | 0.557999607-0.97077818 |
| *SLC25A10* | 0.018077703 | 0.742607445 | 0.580241006-0.950408212 |
| *FAS* | 0.031755499 | 0.756527229 | 0.58644838-0.975931502 |
| *GNLY* | 0.007615009 | 0.766921867 | 0.631115197-0.931952126 |
| *AURKA* | 0.003009852 | 0.769516273 | 0.647213906-0.914929808 |
| *TNFSF10* | 0.006609165 | 0.774810067 | 0.644513794-0.931447311 |
| *HOOK1* | 0.010439899 | 0.784994155 | 0.65223417-0.944776972 |
| *KRT19* | 2.89E-05 | 0.786808819 | 0.703176121-0.880388425 |
| *KRT8* | 8.13E-05 | 0.791891396 | 0.705119704-0.88934117 |
| *BIRC3* | 0.013736486 | 0.801806698 | 0.672613218-0.955815264 |
| *KRT18* | 0.009468551 | 0.804078006 | 0.681964663-0.948057098 |
| *CXCL1* | 0.006207455 | 0.852824105 | 0.760923581-0.955823913 |
| *CXCL5* | 0.025818102 | 0.917321908 | 0.850288195-0.989640322 |
| *FLNC* | 3.92E-06 | 1.230049996 | 1.12652118-1.34309325 |
| *TXNIP* | 0.019103354 | 1.243965136 | 1.036374057-1.49313778 |
| *C5* | 0.038446476 | 1.251746697 | 1.012014012-1.548268872 |
| *C7* | 4.63E-08 | 1.252213745 | 1.155169559-1.357410478 |
| *UCHL1* | 0.000700902 | 1.255176939 | 1.100587612-1.431479992 |
| *LRP1* | 0.049130996 | 1.283313539 | 1.000949968-1.645330627 |
| *EGR1* | 0.00274287 | 1.301878273 | 1.095465085-1.5471849 |
| *NOX4* | 0.007744923 | 1.323470232 | 1.076793589-1.626656651 |
| *CADM1* | 0.000895684 | 1.33173436 | 1.124601007-1.577018332 |
| *LIMCH1* | 0.010906357 | 1.336054401 | 1.068932058-1.669929673 |
| *PPP1R12C* | 0.039418796 | 1.338250505 | 1.014220592-1.765803642 |
| *PALMD* | 0.029852853 | 1.344984949 | 1.02936099-1.757385924 |
| *BNIP3* | 0.000353232 | 1.355866605 | 1.147320541-1.602319653 |
| *SQSTM1* | 0.036506023 | 1.357586478 | 1.019376773-1.80800769 |
| *BCL2* | 0.039156213 | 1.380940957 | 1.016181782-1.876630699 |
| *TPM2* | 8.07E-08 | 1.385897368 | 1.230152939-1.561359936 |
| *ACTC1* | 9.59E-07 | 1.440438658 | 1.244787873-1.666841052 |
| *TPM1* | 1.30E-05 | 1.440637608 | 1.22260953-1.697546654 |
| *FAM83D* | 4.26E-06 | 1.445088055 | 1.235221059-1.690611953 |
| *GSN* | 0.000307115 | 1.458718638 | 1.188304998-1.790668279 |
| *SRRM2* | 0.007857949 | 1.459115625 | 1.104327538-1.927886732 |
| *DIRAS3* | 0.00048717 | 1.463618544 | 1.181581357-1.812976508 |
| *TRPC6* | 0.028338769 | 1.466808742 | 1.041477541-2.065841847 |
| *PRPS1* | 0.036416326 | 1.469982991 | 1.024656881-2.108852275 |
| *MAP1B* | 3.11E-08 | 1.475383846 | 1.285564123-1.693231363 |
| *SOAT1* | 0.01804999 | 1.492449004 | 1.070921293-2.079895175 |
| *LEF1* | 0.000666282 | 1.500050432 | 1.18762704-1.894661559 |
| *CERK* | 0.007300612 | 1.50105124 | 1.115648548-2.019591948 |
| *TRA2A* | 0.041254437 | 1.501656198 | 1.016266363-2.218878257 |
| *AXL* | 0.000214897 | 1.502816603 | 1.211201278-1.864642799 |
| *GPX4* | 0.012665192 | 1.506039967 | 1.091506764-2.078004881 |
| *SVIL* | 1.57E-07 | 1.507939681 | 1.29334726-1.758137317 |
| *ZKSCAN4* | 0.046355029 | 1.511296511 | 1.006693592-2.268830519 |
| *VIM* | 2.15E-05 | 1.515561536 | 1.251020616-1.8360423 |
| *PDLIM7* | 0.000219745 | 1.518973762 | 1.216896336-1.896037668 |
| *SIRT2* | 0.022711327 | 1.521740874 | 1.06041185-2.183769719 |
| *MYL6B* | 0.001975947 | 1.525299252 | 1.167341858-1.993021831 |
| *TNFRSF1A* | 0.02761791 | 1.52530931 | 1.04762006-2.220813232 |
| *LGALS1* | 3.79E-06 | 1.5500762 | 1.2871914-1.86665031 |
| *KL* | 0.000193787 | 1.555172841 | 1.232892194-1.961698336 |
| *OGT* | 0.003607045 | 1.569812467 | 1.158690353-2.126807369 |
| *FUS* | 0.039564337 | 1.570561407 | 1.021794978-2.414048988 |
| *PRKAA2* | 1.69E-06 | 1.602158312 | 1.320999212-1.943158809 |
| *MYO5A* | 0.004153032 | 1.603645181 | 1.161063556-2.214932897 |
| *ULK1* | 0.000332325 | 1.613370042 | 1.242454487-2.095016694 |
| *HGF* | 0.008994751 | 1.614694326 | 1.127096077-2.313234708 |
| *DDX5* | 0.015206812 | 1.624609724 | 1.097954464-2.403885445 |
| *HTRA2* | 0.02895123 | 1.629787326 | 1.051413595-2.526319559 |
| *CNBP* | 0.029859955 | 1.630216666 | 1.048853657-2.533820006 |
| *SOX17* | 0.000297394 | 1.631541339 | 1.251451957-2.12707098 |
| *APOOL* | 0.010705202 | 1.638643402 | 1.121423069-2.394414984 |
| *HNRNPA1* | 0.014195248 | 1.670540376 | 1.108512861-2.517521669 |
| *AHSG* | 0.007237789 | 1.683589334 | 1.151155779-2.462284511 |
| *FLOT1* | 0.002433441 | 1.712244464 | 1.209365909-2.42422999 |
| *LTBP1* | 1.86E-07 | 1.728944745 | 1.407287111-2.124122297 |
| *HSPBAP1* | 0.009770176 | 1.732437022 | 1.141894261-2.628385252 |
| *GLTP* | 0.006364621 | 1.744275394 | 1.169628755-2.601249872 |
| *TUBB6* | 9.44E-10 | 1.767186202 | 1.472553-2.120770575 |
| *SF1* | 0.010038406 | 1.772563935 | 1.146414872-2.740703194 |
| *PABPN1* | 0.000563263 | 1.804450007 | 1.290207677-2.5236556 |
| *RAI14* | 2.35E-07 | 1.835792391 | 1.458122101-2.311283603 |
| *TNRC6B* | 0.001993314 | 1.862318463 | 1.255534694-2.762353023 |
| *MGA* | 0.005088521 | 1.88252829 | 1.209262104-2.930640721 |
| *IQSEC1* | 2.71E-05 | 1.90913449 | 1.411507872-2.582199203 |
| *PINK1* | 5.09E-05 | 1.9150563 | 1.398530528-2.622352933 |
| *RPS27* | 0.002116078 | 1.972963564 | 1.279141215-3.043123916 |
| *MYL6* | 0.000417473 | 2.091648306 | 1.388296543-3.151338709 |
| *RPLP1* | 0.004647396 | 2.097274692 | 1.255820628-3.502539323 |
| *SRP14* | 0.002553948 | 2.114531359 | 1.299969859-3.439497337 |
| *TGFBR1* | 1.10E-06 | 2.17946426 | 1.59317697-2.981504598 |
| *LAMP2* | 8.28E-05 | 2.208600207 | 1.488563892-3.276926774 |
| *DDX17* | 1.62E-07 | 2.276869545 | 1.673542516-3.09770136 |

| GSE26901 | | | |
| --- | --- | --- | --- |
| Symbol | P-Value | HR-Value | 95%CI |
| *AIFM1* | 7.09E-05 | 0.273036713 | 0.143918446-0.517995078 |
| *SLC25A5* | 5.82E-05 | 0.31994374 | 0.183557342-0.557667677 |
| *RPS24* | 0.003820855 | 0.344658236 | 0.167467871-0.709325908 |
| *PNKD* | 0.000620848 | 0.381671018 | 0.219848638-0.662604816 |
| *EZH2* | 0.001529154 | 0.390944982 | 0.218702945-0.698838228 |
| *RFWD3* | 0.003050672 | 0.397730135 | 0.216116238-0.731963789 |
| *COPB2* | 0.028664129 | 0.422739738 | 0.195491901-0.91414982 |
| *IKBKE* | 0.001196871 | 0.441032671 | 0.268767378-0.72371066 |
| *WDR77* | 0.015466984 | 0.441429165 | 0.227710095-0.855735922 |
| *PRDX1* | 0.02435379 | 0.463134536 | 0.236973902-0.90513595 |
| *PABPC1* | 0.016991416 | 0.464605711 | 0.247580079-0.871873324 |
| *KIF11* | 0.000986724 | 0.469129362 | 0.299037222-0.735969779 |
| *RPL34* | 0.009114283 | 0.474992887 | 0.271447947-0.831165775 |
| *GSK3B* | 0.031741813 | 0.491763353 | 0.257305913-0.93985868 |
| *PLK1* | 0.019212004 | 0.494646109 | 0.274404073-0.891658679 |
| *RPL29* | 0.001109804 | 0.502546707 | 0.332337698-0.759929417 |
| *EIF2AK3* | 0.031551624 | 0.504442911 | 0.270332256-0.941295924 |
| *UHRF1* | 0.002293016 | 0.513774733 | 0.334869275-0.788261259 |
| *MRPS12* | 0.049023683 | 0.518197952 | 0.269285458-0.997191303 |
| *RPL23A* | 0.022354824 | 0.529897365 | 0.307285802-0.913778689 |
| *AURKA* | 0.000108688 | 0.530792627 | 0.385145453-0.731517951 |
| *CASP6* | 0.021286499 | 0.551057355 | 0.331840813-0.915089996 |
| *SLC25A10* | 0.004869078 | 0.55295198 | 0.366073215-0.835231531 |
| *CLINT1* | 0.042920983 | 0.555083972 | 0.313952471-0.981416756 |
| *TICAM1* | 0.041186498 | 0.57065095 | 0.333033854-0.977806017 |
| *IDH1* | 0.010232203 | 0.590487954 | 0.394989326-0.882747964 |
| *SLC25A13* | 0.027421167 | 0.590846081 | 0.370158246-0.943107697 |
| *PDIA4* | 0.015694787 | 0.593380157 | 0.388549291-0.906191361 |
| *CTSH* | 0.003449959 | 0.594532178 | 0.419595798-0.842402409 |
| *BUB1B* | 0.040967019 | 0.600479481 | 0.368204331-0.979281277 |
| *KRT18* | 0.034040607 | 0.626058834 | 0.406019797-0.965346187 |
| *SLC25A1* | 0.02852081 | 0.629431613 | 0.415929079-0.952528148 |
| *HOOK1* | 0.009984277 | 0.650313405 | 0.468766622-0.90217073 |
| *XBP1* | 0.036976365 | 0.663319391 | 0.451043983-0.975498245 |
| *ZBP1* | 0.0329645 | 0.663543435 | 0.455147399-0.96735671 |
| *PKP2* | 0.047270487 | 0.689568519 | 0.477639148-0.995531342 |
| *KRT19* | 0.011484247 | 0.789163778 | 0.656792583-0.948213309 |
| *C7* | 0.007480333 | 1.250962888 | 1.061658632-1.474021968 |
| *FLNC* | 0.003936315 | 1.288376844 | 1.084520159-1.530552363 |
| *FAM83D* | 0.019881957 | 1.321377091 | 1.045100635-1.670688313 |
| *TPM2* | 0.00607852 | 1.337671582 | 1.086639715-1.64669599 |
| *SVIL* | 0.01098737 | 1.383165373 | 1.077215566-1.776010773 |
| *ACTC1* | 0.013891531 | 1.398589477 | 1.070573823-1.827106626 |
| *CCL2* | 0.009736829 | 1.409953039 | 1.086621674-1.829493759 |
| *TUBB6* | 0.011159353 | 1.443942538 | 1.08723099-1.917688212 |
| *VIM* | 0.047736971 | 1.455087007 | 1.003746567-2.109375282 |
| *FPR1* | 0.008066357 | 1.459188174 | 1.103316444-1.929845367 |
| *TPM1* | 0.003244234 | 1.487056968 | 1.141786714-1.936735117 |
| *MAP1B* | 0.004017585 | 1.516641932 | 1.141948369-2.014279115 |
| *CADM1* | 0.006825092 | 1.572977091 | 1.132913627-2.183976667 |
| *LGALS1* | 0.004107969 | 1.574332827 | 1.154741457-2.146388556 |
| *ANXA1* | 0.001350954 | 1.597041281 | 1.199440885-2.126441481 |
| *ADAMTSL4* | 0.010793519 | 1.623900911 | 1.118605868-2.357447107 |
| *MYO1B* | 0.030945198 | 1.626515252 | 1.045603302-2.530167855 |
| *FAP* | 0.000436402 | 1.631861781 | 1.242108742-2.143912833 |
| *ZFP36* | 0.003893859 | 1.653808897 | 1.17527205-2.327192131 |
| *TNFRSF10B* | 0.044964126 | 1.656280912 | 1.01139564-2.712357412 |
| *HMOX1* | 0.001591469 | 1.663453133 | 1.212887608-2.281395494 |
| *AXL* | 0.00125524 | 1.729473024 | 1.239851445-2.412447841 |
| *SOX17* | 0.007595726 | 1.73649275 | 1.157992071-2.603996302 |
| *KL* | 0.009463067 | 1.752949043 | 1.147212341-2.678519259 |
| *LIMCH1* | 0.002773653 | 1.786167402 | 1.221471319-2.611927058 |
| *NLRP3* | 0.007560108 | 1.816369168 | 1.172220835-2.814484145 |
| *PLEKHA5* | 0.010302465 | 1.836313327 | 1.154250702-2.921416143 |
| *LRP1* | 0.003041753 | 1.870998863 | 1.236323137-2.831490119 |
| *MYH9* | 0.006000082 | 1.969878522 | 1.214558666-3.194922981 |
| *YWHAG* | 0.042827398 | 2.006345286 | 1.022747663-3.935889129 |
| *RCN1* | 0.006641492 | 2.014286167 | 1.214840595-3.339819873 |
| *RAI14* | 0.000423415 | 2.068031181 | 1.380714032-3.097493666 |
| *MYL6* | 0.045027456 | 2.069423216 | 1.016252933-4.214022226 |
| *TNFRSF1A* | 0.012828517 | 2.159078052 | 1.177592845-3.958599152 |
| *FLOT1* | 0.014043028 | 2.172305891 | 1.169675047-4.034379372 |
| *NOX4* | 8.41E-06 | 2.244433398 | 1.572597395-3.20328731 |
| *RPL27* | 0.03633385 | 2.441733502 | 1.058455874-5.632792679 |
| *RPLP1* | 0.029071466 | 2.782881385 | 1.110005964-6.976925396 |
| *STAT3* | 0.00552892 | 2.81610429 | 1.35520774-5.851828571 |
| *CALU* | 0.000372881 | 2.867728979 | 1.605234334-5.123158235 |
| *CUL4A* | 0.009778528 | 2.884973756 | 1.291413686-6.444932142 |

| GSE15459 | | | |
| --- | --- | --- | --- |
| Symbol | P-Value | HR-Value | 95%CI |
| *SIRT3* | 0.00199885 | 0.330912526 | 0.164100768-0.667291822 |
| *UBE2D3* | 0.006553227 | 0.341066421 | 0.157057132-0.740662343 |
| *NUP214* | 0.001889022 | 0.360992925 | 0.189833286-0.686475456 |
| *IKBKG* | 0.000639521 | 0.377540643 | 0.21583395-0.66040091 |
| *RPS10* | 0.033477568 | 0.395909937 | 0.16852856-0.930077837 |
| *SIRT2* | 0.001085596 | 0.405568956 | 0.236024446-0.696903144 |
| *SLC30A9* | 0.00076366 | 0.405666541 | 0.239876939-0.686040697 |
| *GSK3A* | 0.008633154 | 0.418736576 | 0.218673545-0.801836 |
| *PPM1B* | 0.002985213 | 0.421074394 | 0.237904931-0.745270998 |
| *SERBP1* | 0.008798259 | 0.423958676 | 0.223105726-0.805631315 |
| *ZSCAN20* | 0.023961243 | 0.435404407 | 0.211550424-0.896131495 |
| *CCT8* | 0.011733545 | 0.467455274 | 0.258750902-0.844497282 |
| *ITPK1* | 0.001369408 | 0.484271113 | 0.310649363-0.75492996 |
| *RPS4X* | 0.019604206 | 0.487008399 | 0.266151334-0.891136544 |
| *TRPM7* | 0.019998772 | 0.491104761 | 0.269767003-0.89404517 |
| *RIPK3* | 0.000578151 | 0.494132316 | 0.330744464-0.738233809 |
| *EIF2AK3* | 0.003566676 | 0.498658763 | 0.312287915-0.796254193 |
| *RPS14* | 0.027939104 | 0.500803692 | 0.270319357-0.927807543 |
| *UBR2* | 0.00797623 | 0.504376609 | 0.304203742-0.836267702 |
| *AIFM1* | 0.008228465 | 0.508609591 | 0.308043149-0.839764549 |
| *TET2* | 0.047276516 | 0.511933284 | 0.264193872-0.991982459 |
| *G3BP2* | 0.03217118 | 0.512437487 | 0.277966261-0.944690832 |
| *LRRFIP2* | 0.028027537 | 0.513831982 | 0.283681057-0.930704745 |
| *STUB1* | 0.0059805 | 0.531160205 | 0.338304554-0.833956151 |
| *COPB2* | 0.040341538 | 0.535740949 | 0.295014695-0.972895145 |
| *RIPK1* | 0.041167891 | 0.53700369 | 0.295652526-0.975377975 |
| *TUFM* | 0.023598642 | 0.546123145 | 0.323460136-0.922062587 |
| *ZKSCAN4* | 0.012836835 | 0.552887175 | 0.346669481-0.88177427 |
| *SEC16A* | 0.005429508 | 0.555895563 | 0.367478736-0.84091907 |
| *RPL38* | 0.045511641 | 0.558711508 | 0.315807895-0.988444413 |
| *CASP10* | 0.012105024 | 0.560237513 | 0.356297317-0.880910566 |
| *PELI1* | 0.02224382 | 0.56861722 | 0.35044891-0.922603934 |
| *PDCD6IP* | 0.049695458 | 0.57205756 | 0.327493201-0.999256933 |
| *AFG3L2* | 0.017249965 | 0.572243955 | 0.361458041-0.905950639 |
| *HMGB1* | 0.018221943 | 0.573754831 | 0.36177757-0.909936472 |
| *KRT2* | 0.009715175 | 0.58816222 | 0.393353273-0.879450664 |
| *ATP2A1* | 0.036264159 | 0.589618933 | 0.359603287-0.966761148 |
| *RNF31* | 0.030216235 | 0.603377746 | 0.382088113-0.952829183 |
| *PABPN1* | 0.033927982 | 0.606311072 | 0.38184121-0.962738191 |
| *SHARPIN* | 0.023193801 | 0.609222095 | 0.397159703-0.934514653 |
| *MIB2* | 0.00816852 | 0.620379301 | 0.435528164-0.883686771 |
| *CERK* | 0.011248113 | 0.622974543 | 0.432071959-0.898223717 |
| *PCM1* | 0.028129028 | 0.626096515 | 0.412190215-0.951009585 |
| *CLINT1* | 0.022314621 | 0.627125139 | 0.420273488-0.935785748 |
| *RPL34* | 0.02705571 | 0.631941628 | 0.420689869-0.949274634 |
| *IKBKE* | 0.018005103 | 0.648414297 | 0.452857541-0.928418018 |
| *MAPK3* | 0.017698792 | 0.651282061 | 0.456960856-0.928237764 |
| *SLC25A37* | 0.019782214 | 0.663980903 | 0.47052684-0.936972351 |
| *XBP1* | 0.005890702 | 0.664470725 | 0.496737166-0.888842984 |
| *CASP6* | 0.041885956 | 0.667449855 | 0.452149945-0.985268967 |
| *IDH2* | 0.014781864 | 0.68727312 | 0.508366262-0.929141795 |
| *RPL29* | 0.04853086 | 0.692096604 | 0.480136319-0.997628569 |
| *AKNA* | 0.030812519 | 0.697155298 | 0.502497995-0.967218803 |
| *PNKD* | 0.033654075 | 0.699085354 | 0.502435461-0.972702704 |
| *NQO1* | 0.049356331 | 0.835015415 | 0.697604965-0.999492232 |
| *FLNC* | 0.023770997 | 1.150322018 | 1.018810127-1.298809965 |
| *CCL2* | 0.024873416 | 1.228012254 | 1.02628688-1.469388457 |
| *IGF2BP3* | 0.013688895 | 1.229741013 | 1.043303562-1.449494676 |
| *SVIL* | 0.024091332 | 1.253831738 | 1.030098167-1.526159425 |
| *UCHL1* | 0.020728022 | 1.259494436 | 1.03583238-1.53145071 |
| *TPM2* | 0.002987114 | 1.26928814 | 1.084418927-1.485673426 |
| *MAP1B* | 0.021274832 | 1.269992454 | 1.036248948-1.556460768 |
| *ANXA1* | 0.024692068 | 1.338439382 | 1.037843719-1.726098012 |
| *TUBB6* | 0.005510161 | 1.370165056 | 1.096962169-1.711410233 |
| *TPM4* | 0.0383567 | 1.424098246 | 1.019139348-1.989969101 |
| *LEF1* | 0.01142683 | 1.4273707 | 1.083400047-1.88054922 |
| *LGALS1* | 0.00435732 | 1.442175396 | 1.1212465-1.854962199 |
| *AXL* | 0.004636726 | 1.470393622 | 1.125966907-1.920178461 |
| *PDLIM7* | 0.002456457 | 1.477928338 | 1.14779373-1.903018038 |
| *CTSD* | 0.012796757 | 1.497407981 | 1.089655794-2.057742156 |
| *CTSB* | 0.012753406 | 1.504793682 | 1.090966545-2.075594378 |
| *HSP90AB1* | 0.049313151 | 1.520485242 | 1.001260554-2.308964795 |
| *TUBA1C* | 0.031938145 | 1.547592614 | 1.038425573-2.306417487 |
| *LTBP1* | 0.002298321 | 1.566487316 | 1.173846681-2.090462538 |
| *LITAF* | 0.021452304 | 1.568973042 | 1.068845442-2.303117281 |
| *PAWR* | 0.029634479 | 1.625785484 | 1.049209289-2.519209911 |
| *MYH9* | 0.03005048 | 1.643305314 | 1.049126467-2.57400079 |
| *MERTK* | 0.020689217 | 1.644800544 | 1.079030641-2.507221505 |
| *FLOT1* | 0.036728224 | 1.649601668 | 1.03134773-2.638475447 |
| *LRP1* | 0.000492868 | 1.667142773 | 1.250607631-2.222411695 |
| *ZNF146* | 0.029515403 | 1.667677521 | 1.052205552-2.643160653 |
| *FAP* | 2.94E-05 | 1.669589686 | 1.312772566-2.123391203 |
| *PANX1* | 0.031525262 | 1.674487152 | 1.046705749-2.678792224 |
| *BIRC2* | 0.041661268 | 1.766826251 | 1.021728218-3.055289016 |
| *TUBA1B* | 0.029059361 | 1.817584471 | 1.062865497-3.108213896 |
| *CALU* | 0.001103342 | 1.854481064 | 1.279673155-2.68748313 |
| *XRN2* | 0.008357877 | 1.934712922 | 1.184699743-3.159546637 |
| *RAI14* | 1.67E-05 | 1.960450795 | 1.442967769-2.663515708 |
| *NOX4* | 1.03E-06 | 2.070515557 | 1.546189118-2.77264574 |
| *RPL27* | 0.04647119 | 2.090639795 | 1.011594664-4.320677945 |
| *SQSTM1* | 0.000392356 | 2.19130623 | 1.420181656-3.381132949 |
| *TGFBR1* | 0.000116009 | 2.331325315 | 1.515923005-3.585325712 |
| *RPL6* | 0.039683079 | 2.508801618 | 1.044360175-6.026738386 |
